# Transcriptional Profiling of Ectoderm Specification to Keratinocyte Fate in Human Embryonic Stem Cells

**DOI:** 10.1371/journal.pone.0122493

**Published:** 2015-04-07

**Authors:** Ana Mafalda Baptista Tadeu, Samantha Lin, Lin Hou, Lisa Chung, Mei Zhong, Hongyu Zhao, Valerie Horsley

**Affiliations:** 1 Yale University, Department of Molecular, Cell and Developmental Biology, New Haven, CT, 06511, United States of America; 2 Yale University, Department of Biostatistics, Yale School of Public Health, New Haven, CT, 06520, United States of America; 3 Yale University, Yale Stem Cell Center, Genomics Facility, New Haven, CT, 06520, United States of America; University of Colorado, Boulder, UNITED STATES

## Abstract

In recent years, several studies have shed light into the processes that regulate epidermal specification and homeostasis. We previously showed that a broad-spectrum γ–secretase inhibitor DAPT promoted early keratinocyte specification in human embryonic stem cells triggered to undergo ectoderm specification. Here, we show that DAPT accelerates human embryonic stem cell differentiation and induces expression of the ectoderm protein AP2. Furthermore, we utilize RNA sequencing to identify several candidate regulators of ectoderm specification including those involved in epithelial and epidermal development in human embryonic stem cells. Genes associated with transcriptional regulation and growth factor activity are significantly enriched upon DAPT treatment during specification of human embryonic stem cells to the ectoderm lineage. The human ectoderm cell signature identified in this study contains several genes expressed in ectodermal and epithelial tissues. Importantly, these genes are also associated with skin disorders and ectodermal defects, providing a platform for understanding the biology of human epidermal keratinocyte development under diseased and homeostatic conditions.

## Introduction

The skin serves as a protective barrier that establishes an organism’s first line of defense against external aggressions such as UV light, microbial pathogens, hazardous substances, mechanical stress, and loss of internal bodily fluids [1, 2]. These essential functions are mediated by the epidermis, the outmost layer of the skin, which establishes a tight barrier by creating a stratified epithelium that is separated from the dermis by a basement membrane. During development, the epidermis derives from the primitive ectoderm, a single layer of epithelial cells that differentiates into epidermal basal keratinocytes [1–3]. These actively proliferating cells can symmetrically divide to laterally expand epidermal growth and asymmetrically divide to form the upper, mature squamous layers of the skin epithelium. Cells within the upmost epidermal layer are sloughed from the skin surface and are continually replaced by differentiating basal keratinocytes moving outward.

During embryogenesis, cells of the surface ectoderm, which cover the entire embryo, express the intermediate filaments Keratin 8 (K8) and K18. Around embryonic day 8.5 a few of these cells become committed to an epidermal keratinocyte fate which is marked by a transition in the expression of K8/K18 to K5 and K14 [1, 2, 4, 5]. The K5/K14 positive basal layer cells initiate a program of stratification and eventually undergo terminal differentiation to form the mature adult epidermis, a process that requires the expression of the transcription factor and keratinocyte marker P63.

The molecular mechanisms that regulate epidermal formation following stratification have been the focus of several studies [1] but the mechanisms that control the initial commitment of surface ectoderm to the epidermal lineage during embryogenesis remain elusive. Our previous work shed light into these earlier stages by identifying an unappreciated step during keratinocyte specification [6]. This stage is characterized by the expression of P63 in pre-epidermal keratinocytes prior to K14 expression in fully committed epidermal keratinocytes [6]. Furthermore, impairing γ-secretase related pathways utilizing N-[N-(3,5-Difluorophenacetyl)-L-alanyl]-S-phenylglycine t-butyl ester (DAPT) in human embryonic stem cells (hESCs) or by genetically deleting presenilin 1 and 2 in the developing murine epidermis promotes P63 expression [6].

In recent years hESCs have been used as a model for the study of lineage specification and differentiation. Protocols for differentiation of hESCs into keratinocyte lineages have been developed and shown to be able to generate surface ectoderm cells [6–9]. These protocols have also been shown to mimic the developmental steps that occur during normal *in vivo* murine surface ectoderm development (6). Therefore, these differentiation protocols can be used to identify novel molecular mechanisms that regulate the transition of the surface ectoderm towards an epidermal fate.

Since our previous studies demonstrated that hESCs treated with DAPT enhanced the formation of epidermal progenitors, we used this γ-secretase inhibitor as a pharmacological tool to identify key regulators of non-neural ectoderm specification using RNA sequencing. Our RNA sequencing screen reveals a new transcriptional gene signature associated with early non-neural ectoderm development and with epidermal specification of hESCs.

## Materials and Methods

### Human embryonic stem cell culture

H1 hESC cells were obtained from WiCell [10]. The cells were cultured on matrigel (BD Biosciences) in mTESR1 medium (Stem Cell Technologies) at 37°C, 5% O_2_ and 5% CO_2_ and passaged every 5–6 days using dispase (Stem Cell Technologies). Ectoderm specification of hESCs was performed according to previously described protocols [7]. Briefly, hESC colonies were incubated for 3 days with 0.5 nM of human recombinant bone morphogenic protein (BMP-4) (R&D Systems). From day 4 to day 10, BMP-4 was removed and cells were incubated in medium supplemented with 10% fetal calf serum (FCS; FCII Hyclone). BMP-4 induced differentiation of hESCs is heterogeneous, resulting in cells expressing genes associated with mesoderm and ectoderm lineages, rather than endoderm, neuroectoderm [6]. The γ-secretase complex was inhibited with 5 μM DAPT (Sigma) in ethanol and replaced daily throughout the duration of the specification protocol. Ethanol does not alter the differentiation process [6]. At the end of the specification protocol, RNA samples were isolated with Trizol (Invitrogen) and RNeasy kit (Qiagen) and submitted for mRNA sequencing.

### Stranded mRNA sequencing

cDNA libraries were prepared according to Illumina’s TruSeq Stranded mRNA Sample Preparation Guide for TruSeq Stranded mRNA LT Sample Prep Kit—Set A (Catalog# RS-122-2101). Briefly, poly-A containing mRNA molecules were purified from 4 ug of total RNA using poly-T oligo-attached magnetic beads then fragmented into small pieces using divalent cations under elevated temperature. The cleaved RNA fragments were copied into first strand cDNA using reverse transcriptase and random primers followed by second strand cDNA synthesis using DNA polymerase I and RNaseH. These cDNA fragments then go through an end repair process using a combination of T4 DNA polymerase, E.coli DNA Pol I large fragment (Klenow polymerase) and T4 polynucleotide kinase. The blunt, phosphorylated ends were treated with Klenow fragment (3’ to 5’ exo minus) and dATP to yield a protruding 3- 'A' base for ligation of Illumina's adapters which have a single 'T' base overhang at the 3’ end. These products were then purified and enriched with 15 cycles of PCR to create the final cDNA library of known strand origin. Agencourt AMPure XP magnetic beads by Beckman Coulter were used at each step of the library making process to purify the desired fragments. The final purified DNA was captured on an Illumina flow cell for cluster generation. Libraries were sequenced on the HiSeq 2000 following the manufacturer's protocols.

### Bioinformatics

Raw reads were mapped to the human reference genome (hg19) with TopHat (version 2.0.6) algorithm [11]. RNA abundance and differential expression was calculated by Cuffinks (version 2.0.2) [12]. Among a total of 23615 genes, we selected 1495 genes having p-values less than 0.05 and fold change greater than 2 (759 genes over-expressed and 734 under-expressed) for pathway analysis. Corresponding gene symbols were used to determine the significant enrichment in pathway maps, diseases, or GO processes using the online tool DAVID [13, 14]. The test was performed by hypergeometric model implemented in MetaCore (www.genego.com).

### Indirect immunofluorescence microscopy

hESCs were fixed using 4% paraformaldehyde (Electron microscopy sciences) for 10 minutes at room temperature followed by a 5 minute permeabilization step with 0.2% triton-X100 (Sigma). The following antibodies and dilutions were used for immunostaining: Keratin 14 (rabbit, 1:1000, gift from J. Segre lab), Keratin 14 (1:500, chicken, gift from J. Segre lab), Keratin 18 (MAB3234, mouse, 1:100, Millipore), P63 (ab97865, rabbit, 1:250, Abcam), P63 (sc-8343, rabbit, 1:200, Santa Cruz Technologies), AP2α (3B5S, mouse, 1:10, Developmental Studies Hybridoma Bank), Sox2 (09–0024, rabbit, 1:100, Stemgent), Oct4 (MAB4401, mouse, 1:300, Millipore). Cells were stained with the appropriate fluorophore conjugated secondary antibody (Invitrogen) and mounted in ProLong Gold anti-fade reagent with DAPI for DNA visualization (Invitrogen). Slides were analyzed using a Zeiss Imager.M1 fluorescent microscope (Zeiss) and images were acquired with a color Axiocam MR3 camera (Zeiss).

### Quantitative reverse transcription-polymerase chain reaction

Real Time PCR was performed as described [15]. Briefly, total RNAs were isolated with Trizol (Invitrogen) and RNeasy kit (QIAGEN) from plated undifferentiated and differentiated hESCs treated with vehicle (ethanol) or DAPT. To generate cDNA, equal amounts of total RNA (500 ng) were added to a reverse transcriptase reaction mix (Stratagene) with oligo-dT(12) as primer. Quantitative real time PCR was conducted with a LightCycler system (Roche Diagnostics, Basel Switzerland) using the LightCycle DNA master SYBR Green kit for 45 cycles. Primers used in these experiments are listed in S3 Table. LightCycler analysis software was used for quantifications. The number of cycles required to reach the crossing point for each sample was used to calculate the amount of each product using the 2^-ΔΔCP^ method. Levels of PCR product were normalized to β-actin mRNA levels.

### Statistics

To determine significance between groups, comparisons were made using Students t tests with GraphPad Prism version for Macintosh (GraphPad Software). For all statistical tests, the 0.05 level of confidence was accepted for statistical significance.

### Ethics statement

All work was approved by the Embryonic Stem Cell Research Oversight (ESCRO) committee of Yale University.

## Results

### Surface ectodermal fate markers are induced in DAPT-treated hESCs undergoing epidermal fate specification

**Fig 1:**
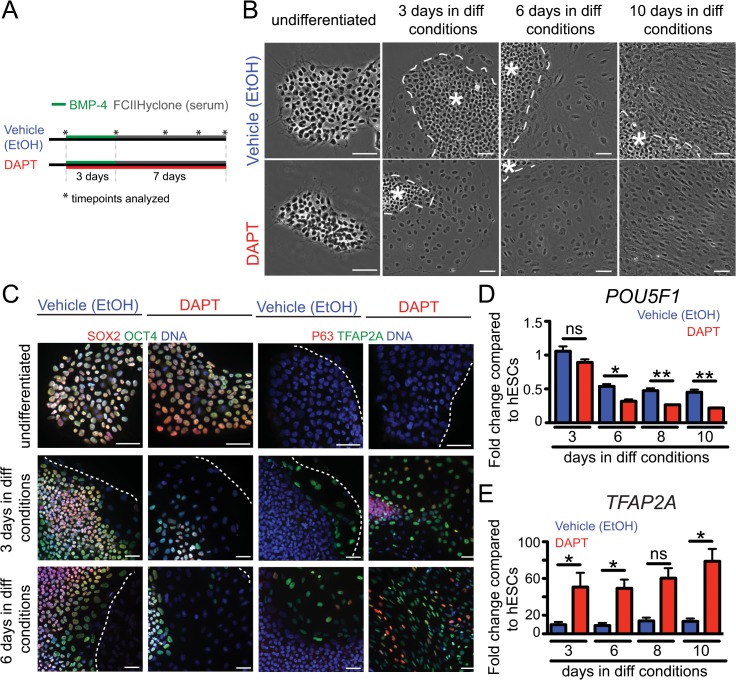
DAPT accelerates ectoderm specification of hESCs. (A) Schematic of the protocol used to induce ectoderm specification of hESCs in the presence of an inhibitor of a **ϒ**-secretase inhibitor (DAPT) or vehicle control (ethanol). (B) Morphological analysis of phase images of hESCs colonies at different timepoints throughout the ectoderm specification protocol. DAPT-treatment accelerates colony differentiation as made evident by a qualitative increase in number of cells displaying a flattened morphological appearance. (C) Undifferentiated hESCs are negative for P63 (red) and AP2α (green), and are positive for the pluripotency markers SOX2 (red) and OCT4 (green). As cells differentiate, DAPT-treated colonies display an increase in P63 and AP2α expression and a loss in OCT4 and SOX2 at 3 and 6 days in differentiation conditions. Dotted line outlines colony edge and the asterisk marks undifferentiated areas of the colonies. (D) Quantitative real-time PCR (qRT-PCR) analysis of mRNA levels of *POU5F1* during differentiation as compared to undifferentiated hESCs (n = 6 independent differentiation experiments for each bar). (E) qRT-PCR analysis of mRNA levels of *TFAP2A* during differentiation as compared to undifferentiated hESCs (n = 3 independent differentiation experiments for each bar). All data are ± SEM (** 0.001<p<0.01, *0.01<p<0.05). Scale bars represent 100μm.

Our previous studies suggest that DAPT treatment of hESCs undergoing epidermal fate commitment enhances the specification of these pluripotent cells towards an epidermal lineage [6]. To analyze early events associated with keratinocyte fate specification, we triggered hESCs to adopt an ectodermal/epidermal lineage via treatment with bone morphogenic protein 4 (BMP-4) and fetal calf serum (FCS) (Fig 1A). Cells were also treated with either a vehicle control (ethanol; not shown to have an effect in the differentiation process [6]) or DAPT for the entire duration of the differentiation protocol. Morphologically, undifferentiated colonies typically have clear borders and round compacted cells characterized by a high nuclear to cytoplasmic ratio and prominent nuclei [10]. The cells at the periphery of the colony often display a flattened appearance with a lower nuclear to cytoplasmic ratio (Fig 1B). In general, colonies containing cells displaying this typical morphology are considered to be differentiating. Based on changes in cell morphology, a qualitative increase in the number of differentiating cells was observed in DAPT-treated cultures versus ethanol controls (Fig 1B). This increase in differentiated cells in DAPT-treated colonies continued throughout the differentiation protocol and was associated with a reduction in the protein and mRNA levels of the pluripotency transcription factors *OCT4 (POU5F1)* and *SOX2*, which are highly expressed in undifferentiated colonies (Fig 1C and 1D). In DAPT-treated cells, we also observed an increase in the number of cells expressing the transcription factor activating enhancer-binding protein 2α (TFAP2A) and tumor protein 63 (TP63), which label surface ectoderm and keratinocyte lineages, respectively (Fig 1C). Analysis of mRNA levels of *POU5F1* and *TFP2A* confirmed the acceleration of differentiation globally in DAPT-treated cells versus vehicle controls (Fig 1D and 1E). A significant increase in the mRNA levels of *TFP2A* started following 3 days of BMP-4 treatment and extended throughout the entire length of the differentiation protocol (Fig 1E). Taken together, these results suggest that differentiation towards a surface ectoderm fate is accelerated in response to DAPT-treated hESCs undergoing ectodermal specification.

### A unique molecular gene signature is associated with ectoderm-specified hESCs

**Fig 2:**
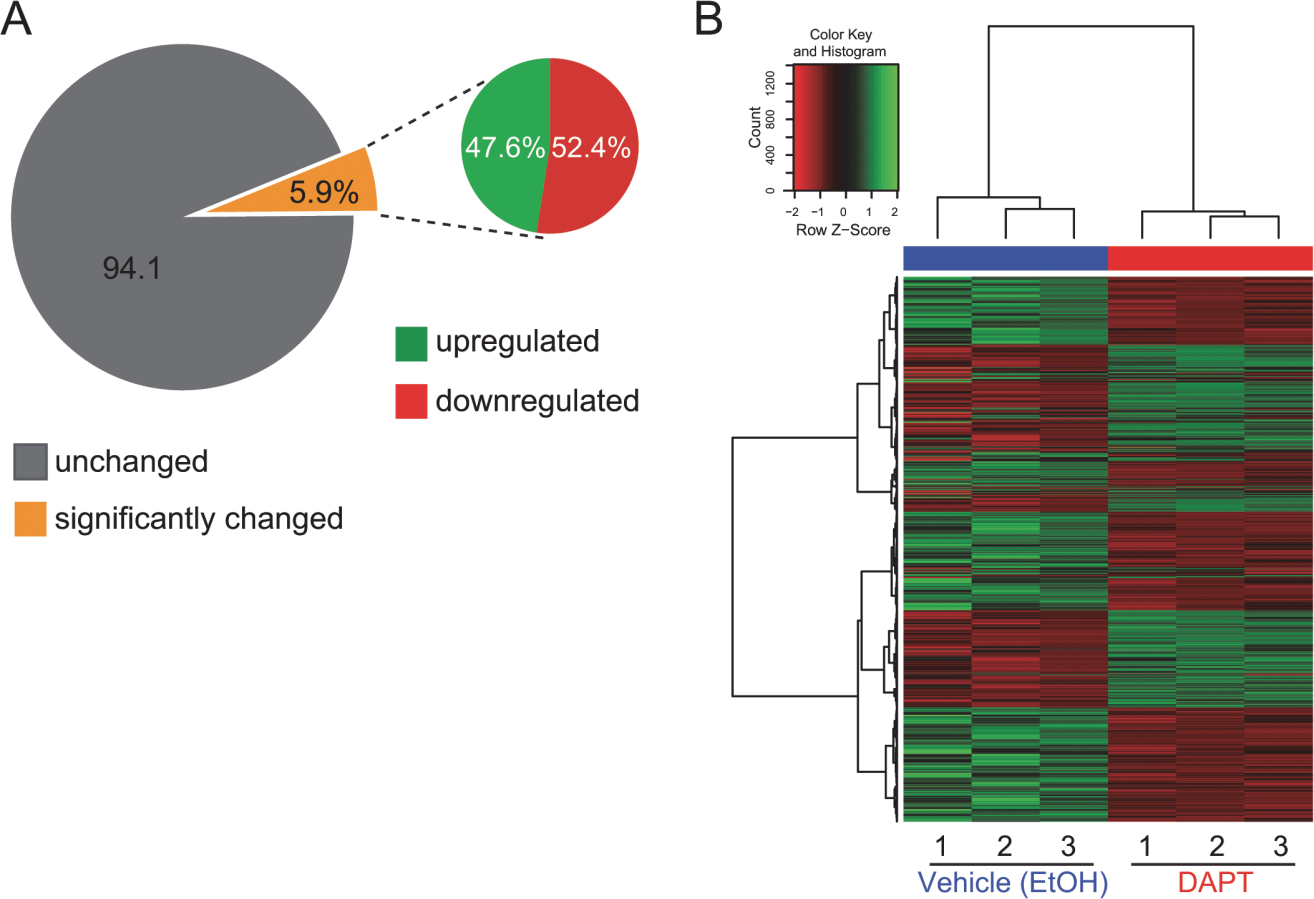
Quantitative analysis of differentially regulated genes in DAPT-treated hESCs during ectoderm specification. (A) Pie chart representation of the percentage of genes that are significantly upregulated and downregulated when hESCs are specified to surface-ectoderm cells in the presence of DAPT compared to vehicle-treated cells. (B) Heat map representation of differentially regulated genes in both the DAPT and vehicle-treated samples (in triplicate). Genes depicted in green correspond to genes that are significantly upregulated in the DAPT-treated sample versus the vehicle-treated samples (log2 fold change >1); genes depicted in red represent genes that are significantly downregulated (log2 fold change <1) in the DAPT-treated sample versus the vehicle-treated control.

To identify the transcriptional changes that occur during early surface ectoderm/keratinocyte specification, we performed transcriptional profiling of ectoderm-specified hESCs treated with either ethanol or DAPT for the entire duration of the differentiation protocol (Fig 1A and S1 and S2 Tables). We chose to compare changes in the transcriptome of control and DAPT-treated hESCs undergoing epidermal specification after 10 days of differentiation, a time point where we have observed the keratinocyte transcription factor *TP63* to be most significantly elevated [7]. Bioinformatic analysis showed that out of more than twenty thousand genes analyzed, roughly 6% of them significantly changed between the vehicle- and DAPT-treated samples (Fig 2A). Out of these, 47.6% of the genes were upregulated in the DAPT-treated sample compared to the vehicle control whereas 52.4% were significantly downregulated (Fig 2A). Hierarchical clustering of the triplicate samples shows the consistency of replicates (Fig 2B).

RNA sequencing results detected alterations in many genes involved in the characterization of both ectodermal cells and epidermal keratinocytes. The sequencing results permitted comparisons of relative expression levels of these genes in the vehicle and DAPT-treated cultures (S1 and S2 Tables). The mRNA levels of several ectoderm and keratinocyte specific genes including *TFAP2A*, *TFAP2B*, *TP63*, *and K5* [16–21] were elevated with DAPT treatment. Conversely, DAPT treatment decreased mRNAs associated with pluripotency, (*OCT4* and *NANOG (22))*, mesoderm [*NK2 homeobox 5* (*NKX2-5)*, *brachyury (T)*, *GATA binding protein 6 (GATA-6)*, *myogenin (MYOG)*, *goosecoid homeobox (GSC)*, *titin (TTN)*, *myosin heavy chain 3 (MYH3) and actin alpha 1 (ACTA1*)], and endoderm [*forkhead box A2 (FOXA2)*, *sex determining region Y-box 17 (SOX17)*, *eomesodermin (EOMES)*, *NODAL*, *mix paired-like homeobox (MIXL)*, *and HNF1 homeobox β (HNF1β)*] [21–25]. Comprehensive lists of the upregulated and downregulated genes are in S1 and S2 Tables.

**Fig 3:**
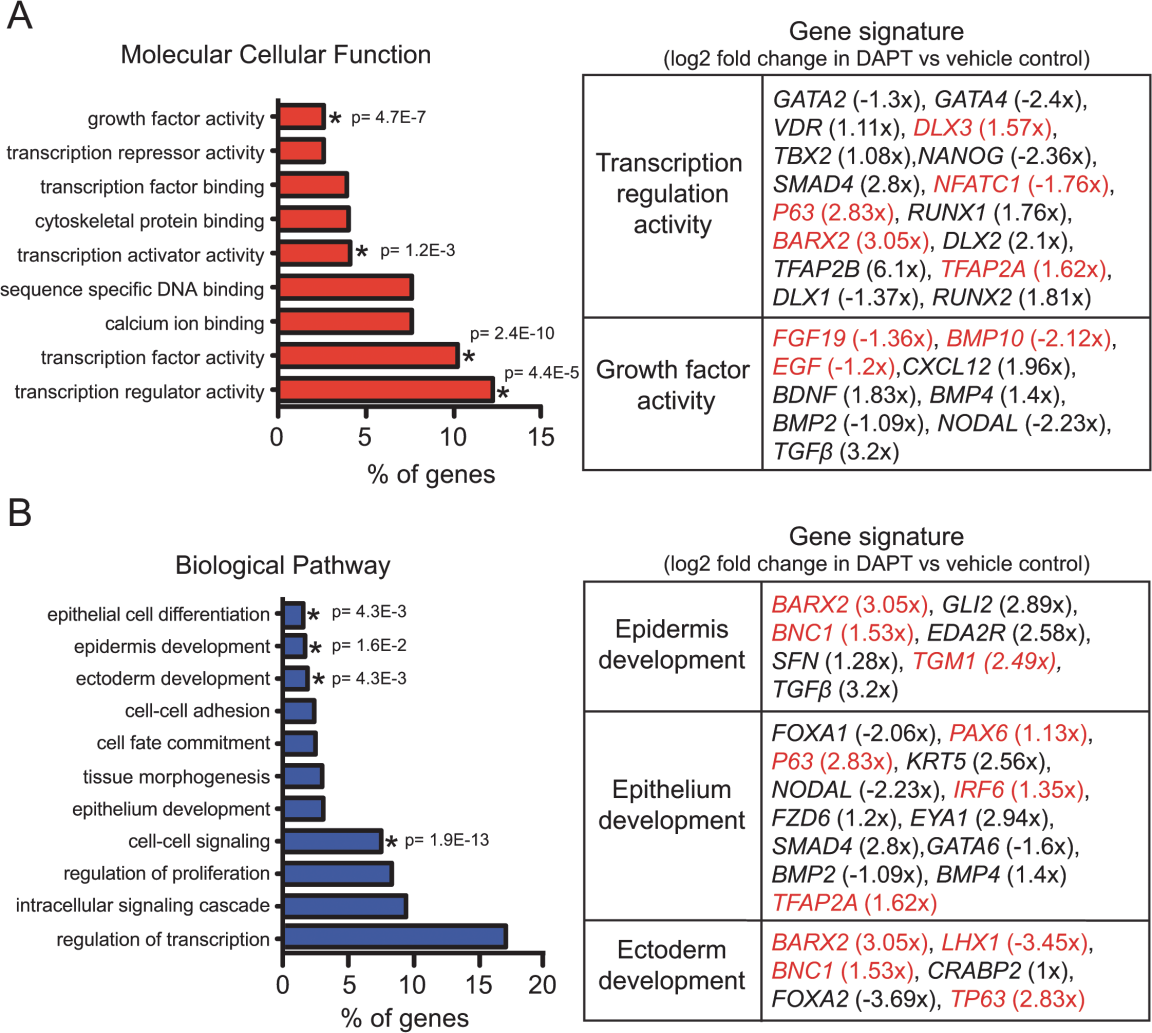
DAPT treatment during ectoderm specification of hESC reveals a transcriptional gene signature associated with ectoderm/epidermal development. (A) Gene ontology analysis reveals a transcription gene signature associated with DAPT treatment during ectoderm specification of hESCs. (B) Gene ontology analysis reveals an upregulation of genes associated with ectoderm, epithelium and epidermal development with DAPT treatment during ectoderm specification of hESCs. The threshold of EASE Score, a modified Fisher Exact P-Value, for gene-enrichment analysis is depicted for specific annotation categories (p value ≤ 0.05 is considered strongly enriched). Genes highlighted in red were corroborated by quantitative real time PCR (see Fig 4).

Given the induction of genes associated with epithelial and epidermal cell fates, we further investigated the classes of genes that change during hESC ectoderm specification in response to DAPT treatment. To this end, we performed gene ontology (GO) analysis using the online DAVID tool to identify significantly enriched categories of genes upregulated within DAPT-treated hESCs specified toward the ectoderm lineage (Fig 3) [13, 14]. Within the genes that significantly changed in the DAPT-treated samples, we found an enrichment of genes associated with transcriptional regulation and growth factor activity (Fig 3A). The epidermis was the primary epithelial lineage associated with genes upregulated with DAPT treatment as indicated by changes in the levels of *K5*, *TP63*, *TFAP2A*, *stratifin* (*SFN)*, *fraser syndrome 1* (*FRAS1)*, *related extracellular matrix protein 2 (FREM2)*, *forkhead box N1 (FOXN1)*, *interferon regulatory 6 (IRF6)* and *basonuclin 1 (BNC1*) [16, 17, 26–34]. Interestingly, several genes associated with mature epidermal cell types including *involucrin* (*IVL*), *transglutaminase 1* (*TGM1*), and *trichohyalin* (*TCHH*) are upregulated in DAPT-treated cultures [35–37]. DAPT-treated ectoderm-specified cells also contained an enrichment of genes associated with the development of a broad spectrum of epithelial lineages including urothelial [*uroplakin 1B* (*UPK1B*, *UPK2*) [38, 39], mammary (*SIX homeobox 1* (*SIX1*)) [40], oral (*ALX homeobox 1*(*ALX1*)) [41] and lung (*eyes absent homologue 1* (*EYA1*)) [42, 43].

**Fig 4:**
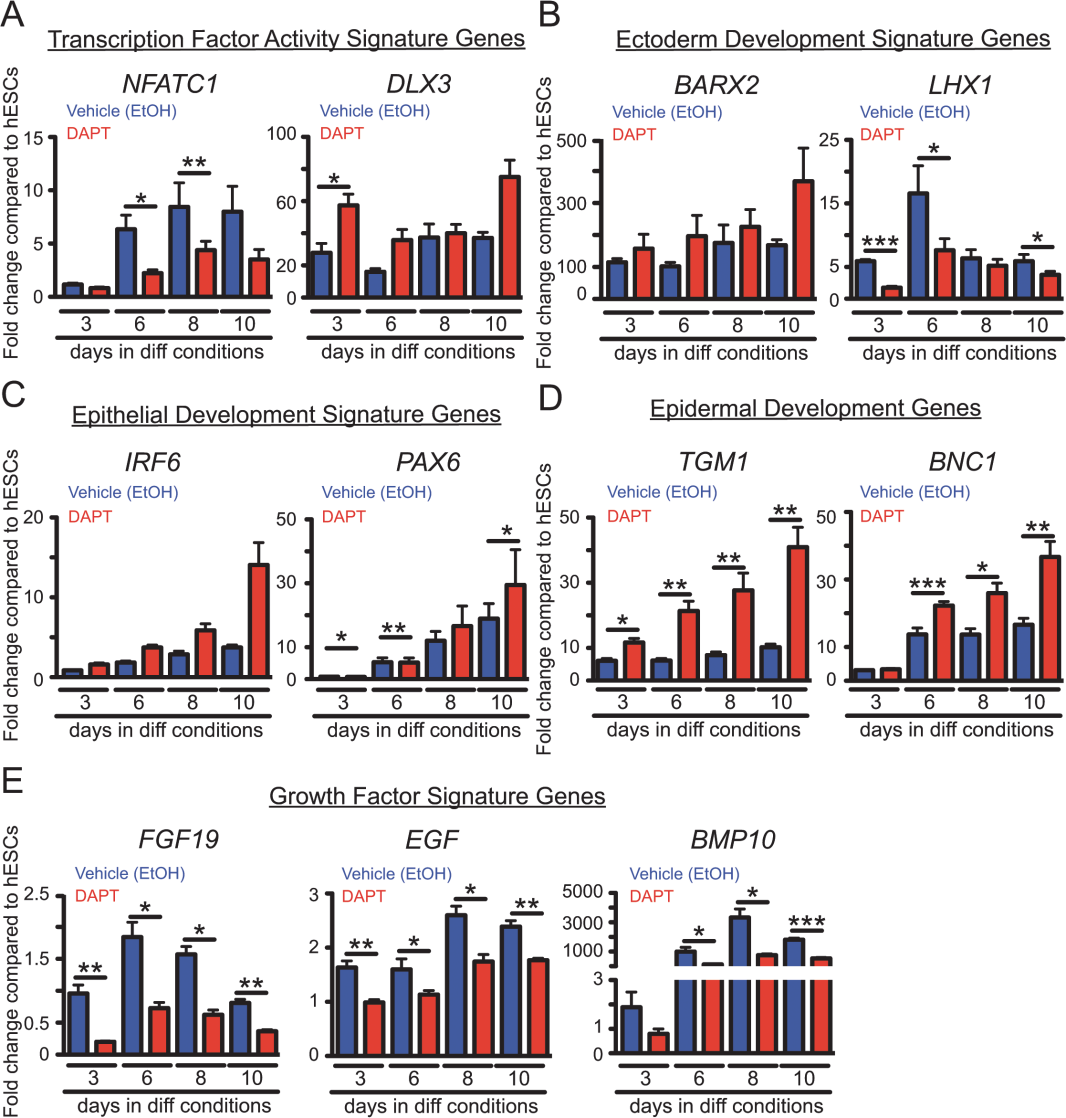
Quantitative real time analysis validates a transcriptional gene signature associated with ectoderm/epidermal development. qRT-PCR analysis of mRNA levels of genes associated with (A) growth factor genes (*FGF19*, *EGF*, *BMP10*), (B) transcriptional regulation (*NFATC1*, *DLX3*), (C) ectoderm development (*BARX2*, *FOXA2*, *LHX1*), (D) epithelial development (*IRF6*, *PAX6*) and (E) epidermal development (*TGM1*, *BNC1*) during the differentiation protocol (see Fig 1A) as compared to undifferentiated hESCs (n = 6 independent differentiation experiments for each bar). All data are ± SEM (*** p<0.001, ** 0.001<p<0.01, *0.01<p<0.05).

Since transcription factor signaling plays an essential role in dictating embryonic stem cell fate [23, 44], we next sought to validate factors involved in guiding hESCs towards an ectodermal/epidermal lineage. Towards this aim, we chose to examine the mRNA levels of genes identified to be relevant in ectodermal/epidermal fate specification as identified by GO analysis (Fig 3) and by previous studies [45–51]. We performed real time PCR on ethanol and DAPT-treated hESCs undergoing a time course of ectodermal/epidermal fate specification. We observed a downregulation of *nuclear factor of activated T-cells* (*NFATC1*) and *LIM Homeobox Protein 1* (*LHX1*) (Fig 4B and 4C). In contrast, the *homeobox proteins BarH-Like 2* (*BARX2*) and *Distal-Less Homeobox 3* (*DLX3*), *IRF6*, and *paired box 6* (*PAX6*) were upregulated (Fig 4C and 4D). The mRNA expression patterns of each of these transcription factors recapitulated trends in fold changes observed in the transcriptional profiling data between ethanol and DAPT-treated cultures, validating our RNA sequencing results. Importantly, this data provides a list of candidate transcription factors that if altered may promote the specification of hESCs towards an ectodermal/epidermal fate.

### Analysis of ectoderm signature expression in mouse embryos and association with human disease

**Fig 5:**
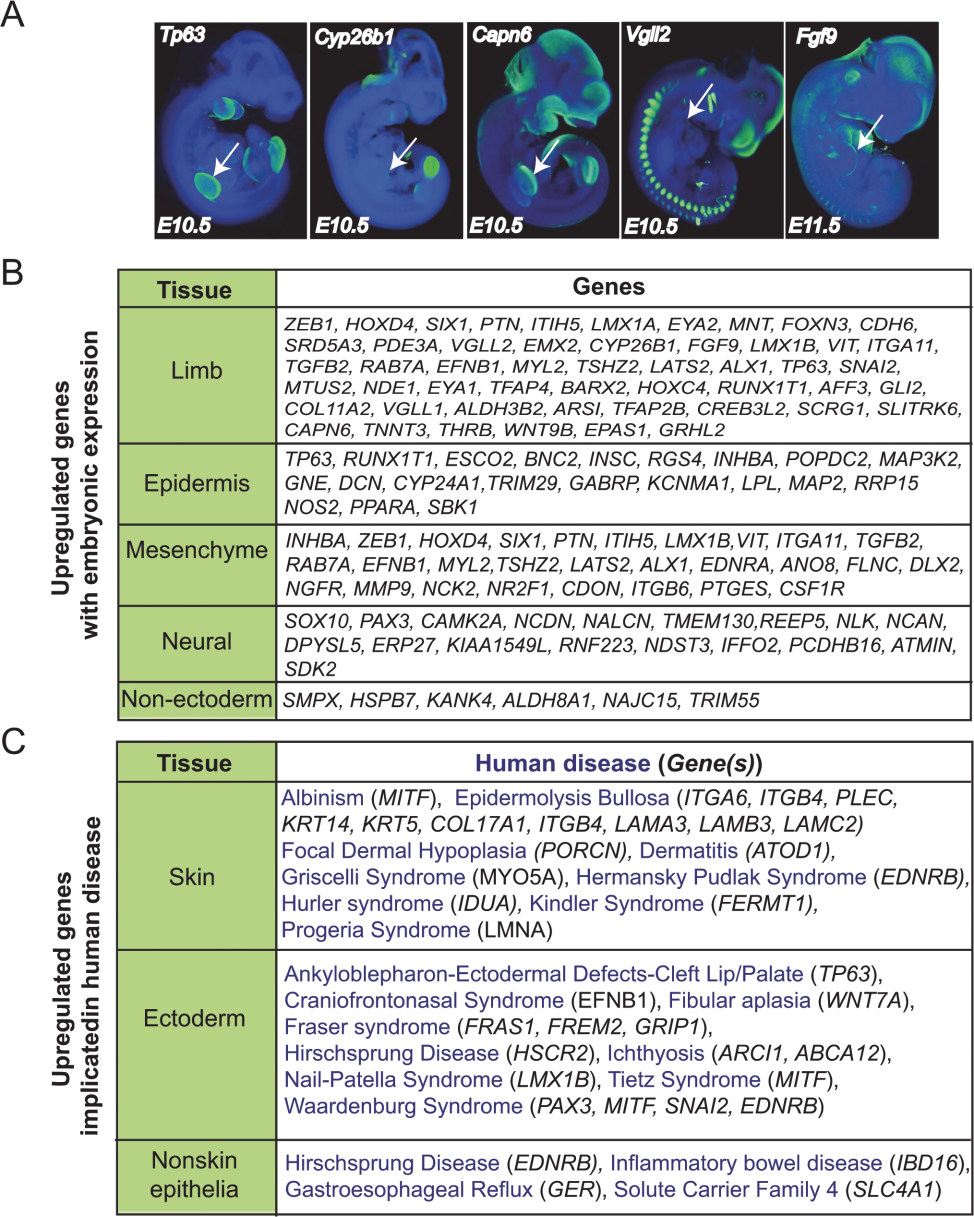
Analysis of in vivo expression and human diseases associated with genes upregulated in DAPT-treated hESCs during ectoderm specification. (A) Images of in situ localization of mRNAs expressed within the murine limb bud at E10.5-E11.5. Data are from the EMAGE gene expression database (EMAGE gene expression database (http://www.emouseatlas.org/emage/)), *P63* EMAGE:5000; *Cyp26b1* EMAGE:5746; *Capn* EMAGE:6170; *Vgll22* EMAGE:6040; *Fgf9* EMAGE:6189. Arrows indicate limb bud localization. (B) Analysis of mRNA expression of 211 upregulated genes with DAPT treatment of ectoderm specified hESCs was performed using EMAGE gene expression database (http://www.emouseatlas.org/emage/) and Gene Expression Database (http://www.informatics.jax.org/expression). (C) Analysis of mRNA expression of 211 upregulated genes with DAPT treatment of ectoderm specified hESCs was performed using the human-mouse connection (http://www.informatics.jax.org/humanDisease.html). Diseases were categorized according to presentation of symptoms in indicated tissues.

To analyze whether genes induced in hESCs by DAPT treatment are expressed in the ectoderm or epithelial lineages in vivo, we analyzed the mRNA expression of genes significantly upregulated by 2 fold using the EMAGE and mouse genomics databases [52] (S4 Table). Of the upregulated genes that displayed expression data, we find that 97% (205/211) of these genes are localized within ectodermal lineages (Fig 5). In particular, several genes display localization similar to p63 in the murine limb bud (Fig 5A and 5B). Interestingly, genes expressed within mesenchymal tissue were present, suggesting the mesenchymal lineages may also be induced by DAPT treatment of ectodermally differentiating hESCs (Fig 5B). Several genes displayed neural expression within the developing embryo, while few of the upregulated genes were expressed in non-ectoderm lineages (Fig 5B). This analysis of mRNA expression confirms that DAPT treatment of ectoderm specified hESCs further enhances specification of ectoderm lineages.

Given the utility of hESCs for analyzing disease phenotypes in particular lineages [53], we analyzed human diseases associated with genes that are upregulated with DAPT treatment of ectoderm specified hESCs (Fig 5C and S5 Table). Several skin and ectodermal disorders were identified including epidermolysis bullosa and cleft palate. Genes involved in non-epidermal diseases were also upregulated by DAPT treatment, supporting the induction of non-epidermal epithelium such as lung. Together, these data support the ability of DAPT to enhance ectoderm specification and provides candidate ectoderm and epidermal diseases that can be studied using hESCs and/or patient-derived iPSCs.

## Discussion

As the body’s largest organ and its first defense against external pathogens, the skin is essential for mammalian survival [1, 2, 54]. Tissue-based replacement therapies utilizing ESC-derived keratinocytes for patients diagnosed with genetic skin diseases like epidermolysis bullosa, burn wounds or chronic ulcers have great potential. However, a major challenge for ESC based cell replacement therapies is the ability to rapidly generate homogeneous cell populations that are specified to a specific lineage for efficient engraftment and are non-tumorigenic [53]. Several groups have made strides in the induction of either induced pluripotent cells or hESCs toward epidermal keratinocyte lineages [6, 8, 17, 34, 55–61]. However, the molecular mechanisms that drive keratinocyte fate specification in embryonic stem cells are not well understood. By taking advantage of the ability of DAPT to enhance epidermal fates during BMP-4/FCS-induced ectoderm specification of hESCs [6], we have determined a unique molecular gene signature for developing human epidermal cells.

The changes in gene expression induced by DAPT treatment of ectoderm specified hESCs supports the enhanced induction of epidermal cell fates [6]. DAPT induces an early adoption of surface ectoderm fate, as indicated by an increase in AP2 transcription factor expression, which is required for normal epidermal development and morphogenesis in *Xenopus* [20] and mice [21, 24, 62]. In addition, *IRF6* was shown to be essential for normal skin, limb and craniofacial morphogenesis [47] and balances proliferation and differentiation during later epidermal specification events [31]. Several other genes that regulate epidermal keratinocyte fate induction such as *TP6*3, *DLX3* [46], *BARX2* [48, 49], *ectodysplasin A receptor* (*EDAR)* [63] and *SFN* [26, 29] were also altered in response to DAPT treatment. We also identified the induction of several genes involved in dictating other epithelial cell fates. For instance, the transcription factor *EYA1* has been previously described as a critical coordinator of epithelial, mesenchymal and vascular morphogenesis in the mammalian lung [29] and been shown to regulate, cell polarity, mitotic spindle orientation and asymmetric division during self-renewal/differentiation of epithelial cells in the embryonic lung distal epithelium [42].

The molecular basis for how these transcriptional regulators influence epidermal fate commitment remains an area of future investigation. It is possible that several of these genes may directly impinge upon the activation of *TP63* expression, which is essential for keratinocyte specification in ESCs [17, 64]. For instance, ChIP-seq experiments have demonstrated that AP2 proteins and TP63 act as co-regulators of epidermal differentiation [65]. Additionally, previous studies indicate that these transcription factors may be involved in regulatory feedback loops to control the expression and activation of TP63. For example, TP63 can induce IRF6 during face morphogenesis [66]. Dlx3 is regulated by p63 during murine ectoderm development [67]. Dlx3 can also post-translationally regulate ΔNp63α protein expression in epithelia [68]. Since TP63 is essential for regulating normal epidermal development and keratinocyte fate in ESCs [17, 64], establishing a mechanistic relationship between these transcription factors and TP63 may be key to defining the signaling events that dictate the progression of hESCs towards an ectodermal/epidermal fate.

Our previous work provided several pieces of evidence that linked Notch signaling to epidermal keratinocyte specification both in mouse embryos and ectoderm specified hESCs [6]. DAPT-mediated inhibition of γ-secretase led to the inactivation of the Notch receptor as indicated by a decrease in Notch intracellular domain (NICD) expression. Importantly, a downregulation in transcript levels for Notch target genes *hes family bHLH transcription factor 5* (*HES5)* corresponded with an increase in p63 transcripts. Our transcriptional profiling data also reveal that *HES5* and *hes-related family bHLH transcription factor with YRPW motif 2 (HEY2)* are downregulated, further supporting that Notch activity was suppressed. However, despite observing decreases in Notch target genes we did not observe alterations in transcript levels for any of the 4 mammalian Notch receptors and several Notch ligands remained expressed following p63 expression. Thus, it is also important to consider that DAPT-mediated induction of ectodermal/epidermal fate specification may be due to other pathways influenced by γ-secretases. For example, several members of the Eph/ephrin family of receptors and ligands including EphA4, EphB2, ephrin-B1 and ephrin-B2 can be proteolytically cleaved by γ-secretases [69–72]. Recent studies have revealed novel roles for Eph/ephrin signaling in regulating adhesion, differentiation and disease in primary human epidermal keratinocytes [73–75], the corneal epithelium [76] and other epithelial tissues [77] which suggests possible roles for these signaling complexes in regulating epidermal fate specification. Indeed, our transcriptional profiling data reveals alterations in the levels of several Eph/ephrin subtypes (S1 and S2 Tables). Future studies using gene silencing approaches or specific pharmacological inhibitors of both Notch-independent and dependent pathways will further our understanding of the molecular mechanisms involved in directing epidermal fate specification.

Our gene signature induced by DAPT in ectoderm specified hESCs displayed expression patterns within the epidermis of developing murine embryos in vivo, further supporting the induction of epidermal genes in DAPT-treated hESC cultures. Interestingly, these analyses also revealed the expression of several genes in the DAPT signature within the mesenchyme of developing embryos, including *LIM homeobox transcription factor 1* (*LMX1)* [78] *and pleiotrophin (PTN)* [79]. Given the importance of mesenchymal-epidermal interactions during development [80], the induction of mesenchymal cells may be required for specification of epidermal fates from hESCs. For instance, *LMX1* expression in the developing mesenchyme induces a dorsal epidermal fate [78]. *PTN* is a mitogen for fibroblast and epithelial cells and has been proposed as a mesenchymal regulator of epithelial embryonic development [79]. Several other genes are expressed in the mesenchyme underlying the ectoderm (Fig 5) and may function to support epidermal fate specification.

Several groups have demonstrated phenotypes from keratinocytes derived from induced-pluripotent stem cells (iPSCs) from patients with genetic skin diseases including genes expressed in our gene signature: type VII collagen (Col7)-deficient recessive dystrophic epidermolysis bullosa (RDEB) [58, 61], *laminin*, *beta 3* gene-deficient junctional epidermolysis bullosa (JEB) [60] and p63 mutant ectodactyly, ectodermal dysplasia, and cleft lip/palate (EEC) syndrome [81]. We identified several additional genes associated with human diseases in our gene signature, providing evidence that this system could be a platform for the use of iPSCs to study skin, craniofacial and other non-epidermal epithelial diseases.

In this study, we have identified a transcriptional gene signature associated with early ectoderm specification of hESCs, providing a relevant resource for the identification of novel players involved in this process. Further analysis of the activity and function of individual genes in this signature will be essential for the better understanding of keratinocyte specification.

## Supporting Information

S1 Table List of upregulated genes detected by transcriptional profiling of ectoderm-specified hESCs treated with DAPT versus Ethanol for 10 days. (DOCX)

S2 Table List of downregulated genes detected by transcriptional profiling of ectoderm-specified hESCs treated with DAPT versus Ethanol for 10 days. (DOCX)

S3 Table List of *Homo sapiens* primers used for qRT-PCR analysis. (DOCX)

S4 Table Tissue localization summary of genes upregulated in response to DAPT treatment using the mouse genomic database. (XLS)

S5 Table Diseases associated with genes upregulated in response to DAPT treatment using the human-mouse connection. (XLS)
