# Supplementary material for: Transcriptional Profiling of Ectoderm Specification to Keratinocyte Fate in Human Embryonic Stem Cells
Source: PLoS One. 2015 Apr 7;10(4):e0122493. doi: 10.1371/journal.pone.0122493 (PMC4388500; doi:10.1371/journal.pone.0122493)
Supplement: S1 Table — (DOCX) [file pone.0122493.s001.docx]

| **S1 Table. List of upregulated genes detected by transcriptional profiling of ectoderm-specified hESCs treated with DAPT versus Ethanol for 10 days.** | | | | | | | | | | | |
| --- | --- | --- | --- | --- | --- | --- | --- | --- | --- | --- | --- |
| **GENE NAME** | **FOLD CHANGE (log2)** | **P-VALUE** | **GENE NAME** | **FOLD CHANGE (log2)** | **P-VALUE** | **GENE NAME** | **FOLD CHANGE (log2)** | **P-VALUE** | **GENE NAME** | **FOLD CHANGE (log2)** | **P-VALUE** |
| *41702* | 3.45308 | 0 | *EPAS1* | 1.88521 | 0 | *LOC646903* | 1.25922 | 0.000307178 | *SEMA5B* | 1.20322 | 0.00014159 |
| *41888* | 1.4874 | 4.5151E-07 | *EPB41L3* | 1.58676 | 5.79536E-14 | *LOC727896* | 2.69309 | 5.11591E-05 | *SENP1* | 2.55132 | 1.98703E-06 |
| *41890* | 1.03862 | 3.69857E-06 | *EPHB1* | 1.23768 | 6.01881E-06 | *LPAR1* | 1.0307 | 0.000652042 | *SERPINB7* | 1.09385 | 0.000640205 |
| *ABCA4* | 1.38881 | 4.25679E-07 | *EPHB2* | 1.18245 | 6.19694E-08 | *LPCAT2* | 1.81514 | 2.22045E-16 | *SERPINB8* | 1.0231 | 0.00255095 |
| *ABCA5* | 1.15597 | 0.00292089 | *EPN3* | 1.48146 | 4.09523E-06 | *LPL* | 2.7287 | 0 | *SERTAD4* | 1.42922 | 2.62609E-07 |
| *ACSL1* | 1.15594 | 7.42884E-06 | *ERO1L* | 2.24422 | 0 | *LPP* | 1.87619 | 0 | *SESN3* | 1.18931 | 1.28652E-08 |
| *ACTBL2* | 1.48323 | 0.000034607 | *ERP27* | 3.90821 | 0 | *LRRC17* | 1.6398 | 9.02385E-07 | *SETDB2* | 2.53253 | 6.54676E-12 |
| *ACVR2A* | 1.82576 | 5.13437E-05 | *ESCO2* | 2.67586 | 0 | *LYRM7* | 3.50449 | 0 | *SFN* | 1.27751 | 1.29634E-08 |
| *ACVR2B* | 2.7657 | 0 | *ETV6* | 1.66724 | 9.45577E-12 | *LYST* | 1.04342 | 0.000499934 | *SFRP1* | 1.35815 | 1.39811E-10 |
| *ADAM28* | 1.86061 | 3.31415E-07 | *EXPH5* | 1.15278 | 1.39315E-05 | *MAML2* | 1.05013 | 3.0731E-06 | *SGMS2* | 1.25101 | 6.99614E-05 |
| *ADAMTS18* | 2.57929 | 0 | *EYA1* | 2.94144 | 1.672E-13 | *MAP1LC3C* | 1.01614 | 0.00345605 | *SH2D4A* | 1.36199 | 4.81621E-08 |
| *ADAMTS6* | 2.68723 | 0 | *EYA2* | 2.80303 | 0 | *MAP2* | 2.93021 | 0 | *SH3KBP1* | 1.15067 | 1.84923E-06 |
| *ADRA1B* | 2.96747 | 0 | *FAM117B* | 2.73424 | 0 | *MAP3K2* | 2.96938 | 0 | *SHB* | 3.27278 | 0 |
| *ADRA2A* | 1.73763 | 0.000138897 | *FAM13B* | 1.02283 | 0.000317796 | *MAPK10* | 2.18573 | 1.9419E-09 | *SIGMAR1* | 1.12862 | 2.49859E-05 |
| *ADRBK2* | 2.27731 | 0 | *FAM177A1* | 2.41036 | 0 | *MAPK4* | 1.43483 | 2.11709E-07 | *SIM2* | 1.13438 | 0.000699841 |
| *AFF2* | 1.26703 | 0.000131319 | *FAM83B* | 1.046 | 4.84342E-05 | *MBNL3* | 1.52052 | 6.16801E-06 | *SIN3A* | 1.31496 | 1.79905E-10 |
| *AFF3* | 4.78895 | 0 | *FAM84A* | 2.71526 | 0 | *MCM8* | 3.02354 | 0 | *SIRPA* | 1.12395 | 2.93315E-06 |
| *AIMP1* | 1.58134 | 1.2803E-09 | *FAM84B* | 1.37109 | 1.14596E-10 | *MDGA1* | 1.78419 | 4.83805E-08 | *SIX1* | 2.32859 | 0.000149642 |
| *AKAP4* | 1.15003 | 0.00448586 | *FBRSL1* | 2.68244 | 0 | *MECOM* | 1.0931 | 0.00591932 | *SLC1A3* | 1.06834 | 1.79015E-05 |
| *AKT3* | 1.23793 | 0.00377594 | *FBXL21* | 3.22389 | 0 | *MEDAG* | 1.20344 | 3.65767E-06 | *SLC1A6* | 1.51933 | 4.05722E-08 |
| *ALDH3B2* | 2.38057 | 0.000242751 | *FERMT1* | 1.90686 | 0 | *MEF2C* | 1.57613 | 3.26702E-06 | *SLC22A5* | 1.41135 | 9.61281E-07 |
| *ALDH8A1* | 2.22555 | 0.00134754 | *FGF9* | 2.07271 | 1.27064E-06 | *MEGF9* | 1.29213 | 9.82832E-07 | *SLC23A2* | 3.64626 | 0 |
| *ALG1* | 1.16705 | 0.0002148 | *FGFRL1* | 1.34918 | 3.20113E-09 | *MEIS1* | 1.4691 | 9.87256E-09 | *SLC24A3* | 1.35727 | 0.000248841 |
| *ALOX5AP* | 1.19675 | 0.00052894 | *FIBIN* | 1.42059 | 3.37362E-08 | *MEIS2* | 1.1817 | 6.70769E-06 | *SLC25A6* | 1.95494 | 0 |
| *ALX1* | 1.99771 | 1.54451E-05 | *FILIP1L* | 1.01019 | 6.72044E-05 | *MET* | 1.79504 | 3.33067E-15 | *SLC27A6* | 2.18208 | 5.10703E-15 |
| *AMER1* | 2.72019 | 0 | *FLNC* | 2.08574 | 2.88658E-15 | *METTL24* | 2.74199 | 4.05651E-11 | *SLC31A1* | 2.38449 | 0 |
| *AMOT* | 1.9535 | 0 | *FLRT2* | 2.04325 | 0 | *METTL7A* | 1.19162 | 1.89876E-07 | *SLC35A3* | 2.00667 | 1.62093E-14 |
| *AMOTL1* | 2.78615 | 0 | *FLVCR1* | 1.71591 | 8.22231E-13 | *MFSD6* | 1.98345 | 5.17586E-13 | *SLC35F2* | 1.04968 | 1.10269E-06 |
| *AMZ1* | 2.13136 | 1.83553E-07 | *FOXN3* | 2.95625 | 0 | *MGC45800* | 1.04293 | 2.13742E-05 | *SLC41A2* | 1.25156 | 1.44892E-05 |
| *ANK1* | 1.38404 | 1.59247E-07 | *FOXP1* | 1.02796 | 0.0010178 | *MINA* | 1.04186 | 7.91171E-05 | *SLC43A3* | 3.04575 | 0 |
| *ANKK1* | 2.2582 | 0.000618826 | *FREM1* | 1.07072 | 1.56914E-05 | *MIR22HG* | 1.30613 | 1.99638E-05 | *SLC44A3* | 1.09172 | 0.00365724 |
| *ANKRD19P* | 1.22942 | 4.21719E-07 | *FREM2* | 1.67327 | 8.88178E-16 | *MKI67* | 1.86262 | 7.26086E-14 | *SLC4A4* | 1.79228 | 1.46725E-11 |
| *ANKRD34B* | 1.13651 | 1.88729E-05 | *FRMD6* | 1.04745 | 4.88134E-06 | *MME* | 1.01604 | 5.21545E-05 | *SLC5A12* | 1.75686 | 1.83157E-08 |
| *ANKRD65* | 1.0737 | 0.00394185 | *FUNDC2* | 2.40528 | 0 | *MMP9* | 1.73615 | 1.13243E-14 | *SLC7A1* | 1.07699 | 2.22897E-07 |
| *ANKS1A* | 1.47341 | 9.16089E-12 | *FZD6* | 1.20475 | 2.22563E-07 | *MN1* | 1.13588 | 1.85035E-05 | *SLITRK6* | 2.57801 | 0 |
| *ANO8* | 1.89254 | 0.000231704 | *GABRP* | 4.73243 | 0 | *MNT* | 2.79379 | 0 | *SLN* | 1.42664 | 1.74324E-08 |
| *ANXA8L2* | 1.52028 | 3.63129E-10 | *GADD45G* | 1.24505 | 0.00294207 | *MTMR11* | 1.56594 | 2.25494E-07 | *SMAD4* | 2.80791 | 0 |
| *ARAP2* | 2.36525 | 0 | *GARNL3* | 1.13253 | 0.000755274 | *MTUS2* | 3.60148 | 0 | *SMAD9* | 1.14011 | 3.73443E-05 |
| *ARHGAP29* | 1.50976 | 4.38094E-13 | *GAS1* | 1.53538 | 1.41236E-08 | *MUC15* | 3.3512 | 0.000224209 | *SMARCA2* | 1.28903 | 6.77664E-06 |
| *ARHGAP31* | 1.0956 | 1.10322E-06 | *GDF6* | 2.47362 | 0 | *MUC4* | 2.17325 | 4.21885E-15 | *SMPX* | 2.52185 | 0.00379028 |
| *ARHGAP35* | 2.40506 | 0 | *GFRA1* | 4.03659 | 0 | *MXD1* | 2.56834 | 0 | *SMTNL2* | 1.16956 | 6.21718E-05 |
| *ARHGEF9* | 2.8757 | 0 | *GLI2* | 2.89558 | 0 | *MXRA5* | 1.3867 | 9.11307E-11 | *SMYD2* | 1.03035 | 4.79839E-06 |
| *ARID3B* | 1.57086 | 6.30607E-14 | *GLIPR1* | 1.48781 | 6.74435E-08 | *MYL2* | 3.15675 | 0.0008799 | *SNAI2* | 3.00907 | 0 |
| *ARNTL* | 2.05798 | 9.51161E-11 | *GLRB* | 1.47823 | 0.000796069 | *MYO5A* | 1.5216 | 8.7641E-12 | *SNAP25* | 3.33779 | 2.45659E-10 |
| *ARRB1* | 1.96064 | 0 | *GLYR1* | 3.11869 | 0 | *MYO5B* | 1.42828 | 2.04733E-10 | *SNAP29* | 3.35699 | 0 |
| *ARSD* | 1.20485 | 2.46851E-06 | *GNAZ* | 1.35809 | 1.02578E-06 | *NALCN* | 2.7679 | 1.42027E-09 | *SNX2* | 3.17268 | 0 |
| *ARSI* | 2.30159 | 0 | *GNE* | 2.72422 | 0 | *NAV1* | 2.98108 | 0 | *SORCS2* | 1.42729 | 6.10262E-07 |
| *ASH1L* | 1.59293 | 5.72625E-10 | *GPATCH2L* | 2.9014 | 0 | *NCALD* | 1.68239 | 1.19065E-11 | *SOWAHA* | 2.41529 | 8.78708E-06 |
| *ATL1* | 1.18594 | 0.00034089 | *GPBP1L1* | 1.6325 | 3.77476E-15 | *NCAM1* | 1.34893 | 3.43544E-05 | *SOX10* | 2.42846 | 6.21725E-15 |
| *ATMIN* | 3.21802 | 0 | *GPR161* | 1.2445 | 1.16344E-08 | *NCAN* | 2.63032 | 0 | *SPATA6* | 2.61632 | 0 |
| *ATP11A* | 1.21739 | 4.00459E-06 | *GPR173* | 2.13677 | 2.22045E-16 | *NCDN* | 2.33541 | 0 | *SPIN1* | 1.27727 | 6.32159E-10 |
| *ATP1B1* | 1.19452 | 4.20752E-08 | *GPR37* | 1.12701 | 1.12471E-06 | *NCF2* | 2.052 | 2.26696E-11 | *SPNS2* | 1.70812 | 8.60237E-06 |
| *ATP2C2* | 1.34632 | 0.000305447 | *GPR56* | 1.91262 | 3.34843E-13 | *NCK2* | 2.58628 | 0 | *SPRY4* | 1.29524 | 1.94783E-09 |
| *ATP6V0A4* | 1.35646 | 0.000213207 | *GPR63* | 1.23507 | 1.37322E-05 | *NDE1* | 2.57379 | 0 | *SRD5A3* | 2.50235 | 0 |
| *BAALC* | 1.38495 | 7.89344E-05 | *GPR68* | 1.37455 | 0.000328341 | *NDNF* | 2.45405 | 0 | *SRGAP2* | 2.11577 | 0 |
| *BACH2* | 1.0394 | 0.00390881 | *GPR85* | 1.65802 | 0.000120703 | *NDST3* | 3.32557 | 2.19553E-10 | *SSBP2* | 1.00353 | 0.00313575 |
| *BARX2* | 3.05742 | 2.22045E-16 | *GPR87* | 1.51752 | 4.94707E-06 | *NET1* | 1.13322 | 5.44116E-08 | *SSFA2* | 1.33672 | 2.33553E-10 |
| *BDNF* | 1.83834 | 1.09717E-07 | *GPRC5A* | 1.38429 | 1.60171E-09 | *NEUROD1* | 7.17367 | 0.00502605 | *SSPN* | 2.70506 | 4.44089E-16 |
| *BEND6* | 1.02876 | 0.00266954 | *GRHL1* | 1.03029 | 0.000269097 | *NFATC2* | 1.45043 | 3.84867E-06 | *STAG3L1* | 1.40802 | 5.15745E-07 |
| *BEND7* | 1.57264 | 1.53212E-08 | *GRHL2* | 1.83284 | 2.22045E-16 | *NFIL3* | 1.14419 | 1.32899E-06 | *STAG3L3* | 1.31669 | 3.12421E-06 |
| *BICC1* | 1.11365 | 0.00151166 | *GTF2H2B* | 2.44424 | 0 | *NGFR* | 1.61782 | 3.1442E-11 | *STAP2* | 1.1728 | 0.000226895 |
| *BMP4* | 1.40534 | 1.67772E-10 | *GTF2IRD2* | 1.14429 | 0.000297331 | *NKX2-6* | 3.31601 | 3.76238E-06 | *STARD13* | 1.37754 | 4.09243E-06 |
| *BMP5* | 1.34523 | 3.23266E-06 | *H2AFJ* | 1.41836 | 4.09348E-08 | *NKX3-1* | 1.3255 | 0.000370409 | *STAT6* | 1.00421 | 0.000165149 |
| *BNC1* | 1.52866 | 5.10147E-12 | *HAPLN1* | 1.1915 | 4.99956E-07 | *NLK* | 3.16442 | 0 | *STS* | 1.75248 | 1.29135E-09 |
| *BNC2* | 2.48242 | 0 | *HAVCR2* | 1.4788 | 1.28515E-05 | *NOS2* | 2.67174 | 4.98268E-12 | *STX1B* | 1.54456 | 7.63955E-08 |
| *BTBD9* | 3.07934 | 0 | *HCG4* | 1.42207 | 0.00288544 | *NOX4* | 2.54853 | 1.66533E-14 | *SULF1* | 1.01748 | 3.34224E-06 |
| *BVES* | 3.04121 | 0 | *HEY1* | 1.3073 | 1.21448E-05 | *NPR3* | 1.37469 | 8.8109E-06 | *SYNPO2* | 2.48049 | 6.48592E-13 |
| *C15orf41* | 2.80062 | 0 | *HIVEP3* | 1.21683 | 0.00266896 | *NR2F1* | 2.66374 | 5.06717E-08 | *TACSTD2* | 1.44185 | 9.24466E-09 |
| *C15orf48* | 1.09709 | 2.86631E-05 | *HMGA2* | 1.39772 | 1.71254E-09 | *NR2F2* | 1.84122 | 1.57852E-12 | *TANC2* | 1.81704 | 0 |
| *C3orf17* | 2.37895 | 0 | *HMGCLL1* | 1.59914 | 0.0026474 | *NRIP1* | 1.35361 | 1.52942E-09 | *TANGO2* | 1.31546 | 1.20996E-07 |
| *C4orf26* | 2.28065 | 2.77369E-11 | *HNMT* | 1.40167 | 0.00117619 | *NRK* | 1.46131 | 1.7152E-10 | *TBX2* | 1.22285 | 0.0019623 |
| *C5* | 1.252 | 4.11074E-06 | *HOXC4* | 3.05682 | 3.81307E-06 | *NRXN3* | 1.49211 | 0.0000266 | *TBX4* | 1.03007 | 0.000171366 |
| *C5orf30* | 1.28877 | 3.97253E-08 | *HOXC9* | 2.27567 | 0.000185215 | *NTRK2* | 1.56444 | 2.4142E-07 | *TENM2* | 1.62159 | 9.08162E-14 |
| *C5orf46* | 1.5087 | 1.23082E-10 | *HOXD4* | 2.71784 | 0.000154776 | *NXPH2* | 2.58215 | 0 | *TEX2* | 1.66425 | 1.33227E-15 |
| *C6orf123* | 2.41072 | 0.00662357 | *HOXD9* | 1.75091 | 0.00289285 | *OLFM3* | 3.21498 | 1.81813E-05 | *TFAP2A* | 1.62006 | 4.01679E-13 |
| *C8orf4* | 1.16285 | 2.61624E-08 | *HPCAL4* | 2.00245 | 7.97041E-06 | *OLFML2A* | 1.21904 | 9.2982E-09 | *TFAP2B* | 6.11374 | 0 |
| *CA3* | 1.22786 | 3.29239E-07 | *HPS3* | 2.23458 | 0 | *OLR1* | 3.06412 | 0 | *TGFB2* | 3.20364 | 0 |
| *CACNA1C* | 1.45995 | 1.81782E-06 | *HPSE2* | 1.73788 | 0.00246599 | *OSBPL9* | 1.78011 | 6.8529E-08 | *TGFBI* | 1.18181 | 9.29595E-06 |
| *CACNA2D2* | 1.39385 | 4.60287E-08 | *HS3ST1* | 1.25779 | 0.000168442 | *OTX1* | 1.37364 | 0.000106151 | *TGM1* | 2.49938 | 0 |
| *CACNG4* | 1.20872 | 2.76321E-08 | *HS3ST4* | 1.56449 | 1.25314E-07 | *OXGR1* | 1.65842 | 3.97144E-05 | *THRB* | 2.11624 | 1.26565E-14 |
| *CALCOCO1* | 1.1801 | 3.41766E-08 | *HSF2BP* | 1.20267 | 0.00191395 | *OXTR* | 3.34081 | 0 | *THSD4* | 1.19878 | 2.16699E-07 |
| *CAMK1G* | 1.23276 | 0.000474068 | *HSPB7* | 1.87486 | 3.85691E-13 | *P2RY2* | 2.77653 | 3.95395E-12 | *TIAM2* | 1.1108 | 0.00186795 |
| *CAMK2A* | 2.46151 | 8.15925E-12 | *HTR1E* | 2.94601 | 4.82281E-13 | *P2RY6* | 2.43376 | 4.26937E-09 | *TMCC3* | 1.64203 | 4.34377E-10 |
| *CAMK2N1* | 3.27855 | 0 | *IFFO2* | 3.14248 | 0 | *PAEP* | 3.21574 | 7.07396E-05 | *TMEM106C* | 1.27241 | 1.06425E-07 |
| *CAPN6* | 2.46425 | 0 | *IFNLR1* | 1.02542 | 0.00183367 | *PAG1* | 1.03577 | 0.000272596 | *TMEM117* | 1.08766 | 0.000461625 |
| *CASC3* | 2.78087 | 0 | *IFT80* | 1.34095 | 5.00005E-06 | *PAPD7* | 1.68212 | 2.43139E-13 | *TMEM150C* | 1.55807 | 3.89825E-07 |
| *CASQ2* | 1.38488 | 0.000409763 | *IGF2* | 1.50886 | 6.60041E-06 | *PAPPA2* | 2.0418 | 5.37459E-12 | *TMEM176A* | 3.45464 | 0.000348451 |
| *CAST* | 1.15493 | 0.000116066 | *IGFBP3* | 1.37111 | 1.85011E-08 | *PATL1* | 1.21384 | 1.41748E-08 | *TMEM176B* | 2.41397 | 8.82192E-05 |
| *CAV1* | 1.19384 | 3.08726E-07 | *IGFBP5* | 1.74862 | 1.5099E-14 | *PAX3* | 2.37712 | 7.99385E-11 | *TMEM178A* | 1.22805 | 1.44317E-05 |
| *CBR3-AS1* | 2.14399 | 0.000184799 | *IGLON5* | 2.49168 | 0 | *PAX6* | 1.13034 | 3.31554E-05 | *TMEM40* | 1.09891 | 8.69781E-06 |
| *CCBE1* | 1.00458 | 0.00019446 | *IKZF2* | 1.17116 | 0.00027298 | *PAX7* | 2.05732 | 0.000104967 | *TMPRSS4* | 2.87205 | 0.00049711 |
| *CCBP2* | 1.65042 | 3.46547E-07 | *IKZF4* | 2.49236 | 0 | *PBRM1* | 1.52985 | 5.10703E-13 | *TMTC2* | 1.45396 | 3.23246E-09 |
| *CCDC3* | 1.03184 | 2.32738E-05 | *IL17RE* | 1.17265 | 0.00138516 | *PCDHB16* | 3.16502 | 0 | *TNC* | 1.62135 | 5.67546E-13 |
| *CCDC85A* | 1.54615 | 2.17137E-06 | *IL1R1* | 1.06693 | 1.69922E-05 | *PCSK1N* | 1.67675 | 8.34215E-05 | *TNFSF15* | 2.57764 | 0 |
| *CCND1* | 2.39487 | 0 | *IL36RN* | 3.13271 | 2.47801E-05 | *PDE1B* | 1.30745 | 9.48481E-05 | *TNNT3* | 2.05397 | 5.84103E-05 |
| *CDH10* | 1.55745 | 1.6438E-10 | *INHA* | 1.5268 | 1.48322E-05 | *PDE3A* | 2.14573 | 4.20552E-12 | *TNRC6B* | 1.15793 | 5.16182E-07 |
| *CDH6* | 2.98761 | 0 | *INHBA* | 2.82991 | 0 | *PDE4D* | 1.84205 | 1.53877E-13 | *TP53I11* | 1.60096 | 2.01143E-08 |
| *CDKN2B* | 2.61278 | 0 | *INPP4B* | 1.75609 | 9.98367E-08 | *PDGFC* | 1.33377 | 3.56845E-09 | *TP63* | 2.82918 | 0 |
| *CDON* | 3.68666 | 0 | *INSC* | 2.63599 | 0.000124966 | *PIK3C2A* | 1.25416 | 2.39449E-09 | *TPM1* | 1.14058 | 0.000193352 |
| *CDS1* | 1.08924 | 6.18717E-06 | *IPO9* | 2.48226 | 0 | *PIK3R1* | 2.13354 | 0 | *TRAF3* | 1.85894 | 1.87137E-11 |
| *CELSR1* | 1.60879 | 5.57554E-13 | *IQCA1* | 1.34549 | 2.76074E-07 | *PKI55* | 1.50395 | 1.08957E-06 | *TRAF3IP2* | 1.76562 | 2.43001E-10 |
| *CHMP3* | 1.05472 | 0.000452155 | *IQCJ-SCHIP1* | 1.27967 | 0.000516207 | *PKNOX2* | 1.73184 | 3.5838E-13 | *TRIL* | 1.66968 | 4.26384E-10 |
| *CHN2* | 2.43625 | 4.88054E-13 | *IRF6* | 1.34913 | 7.88092E-10 | *PLA2R1* | 1.60526 | 5.20412E-08 | *TRIM13* | 1.8435 | 3.57558E-12 |
| *CHORDC1* | 1.84865 | 3.9968E-15 | *IRF8* | 1.26334 | 0.000789331 | *PLAC8* | 1.95811 | 3.88616E-05 | *TRIM2* | 1.03244 | 0.0012709 |
| *CHST11* | 3.65438 | 0 | *IRX1* | 2.79544 | 2.60902E-13 | *PLAG1* | 1.6917 | 1.63301E-09 | *TRIM29* | 2.67875 | 7.21259E-11 |
| *CLEC19A* | 1.70013 | 0.00289011 | *IRX2* | 1.33781 | 1.04251E-06 | *PLCXD3* | 1.25707 | 2.90803E-05 | *TRIM55* | 2.21358 | 0 |
| *CLIC3* | 1.34837 | 0.000172757 | *IRX3* | 1.36697 | 0.00208157 | *PLD5* | 1.68388 | 1.89847E-07 | *TSHZ2* | 2.65006 | 3.33067E-15 |
| *CLMN* | 1.22822 | 2.24322E-07 | *ISL1* | 1.20562 | 1.92514E-06 | *PLEKHA6* | 1.43107 | 9.20868E-08 | *TSNAX-DISC1* | 2.8431 | 0.00474232 |
| *CNTN4* | 1.30064 | 0.00079452 | *ITGA11* | 3.06404 | 0 | *PLEKHA7* | 1.88482 | 6.66134E-16 | *TSPAN2* | 1.61346 | 1.89733E-11 |
| *COL11A2* | 2.58428 | 0.000148683 | *ITGA4* | 1.14294 | 3.95688E-05 | *PLEKHG1* | 1.36027 | 4.24903E-07 | *TTI1* | 1.19697 | 6.93408E-08 |
| *COL12A1* | 1.68205 | 4.8006E-12 | *ITGB4* | 2.2078 | 0 | *PLEKHG4B* | 1.07642 | 1.57162E-06 | *TTLL7* | 1.53144 | 2.7085E-08 |
| *COL17A1* | 2.35134 | 0 | *ITGB6* | 2.60731 | 0 | *PLEKHN1* | 1.61377 | 0.000782524 | *TXNL4B* | 2.43061 | 0.00150184 |
| *COL2A1* | 1.54483 | 1.4817E-12 | *ITIH5* | 2.03986 | 0 | *PLP1* | 1.00976 | 3.03896E-05 | *UBD* | 1.0205 | 0.000125286 |
| *COL8A2* | 1.15735 | 0.000306293 | *JAKMIP2* | 2.34039 | 6.15352E-12 | *PLSCR5* | 4.89902 | 2.61124E-13 | *UBE2J1* | 1.05785 | 4.37781E-07 |
| *COX4I2* | 2.34721 | 8.33161E-10 | *KANK1* | 1.0589 | 1.59925E-06 | *PLXDC2* | 1.32772 | 2.64327E-07 | *UBE2QL1* | 1.64107 | 2.65665E-06 |
| *CPE* | 1.25072 | 2.89128E-09 | *KANK4* | 2.04579 | 0 | *PLXNA2* | 1.09425 | 3.68989E-07 | *UBL3* | 1.17508 | 2.8384E-08 |
| *CPPED1* | 3.01376 | 0 | *KANSL1L* | 2.08266 | 3.10176E-06 | *POPDC2* | 2.91468 | 4.44089E-16 | *UBLCP1* | 3.54646 | 0 |
| *CRH* | 2.83943 | 3.36329E-09 | *KAT6A* | 2.42923 | 0 | *PORCN* | 1.61799 | 2.76328E-07 | *UBN2* | 1.68222 | 1.00719E-12 |
| *CRHBP* | 1.03209 | 3.03259E-06 | *KCNE4* | 1.06128 | 5.43809E-05 | *POU3F2* | 1.68716 | 0.000449284 | *UBQLN4* | 1.11629 | 2.28324E-07 |
| *CRISPLD2* | 1.85671 | 3.77476E-15 | *KCNH1* | 1.09398 | 0.00244144 | *PPARA* | 2.16372 | 0 | *UG0898H09* | 1.26066 | 0.000715376 |
| *CSDC2* | 3.58131 | 0 | *KCNJ15* | 1.65687 | 0.000765173 | *PPP1R13B* | 1.4862 | 3.25168E-08 | *UNC5C* | 1.39238 | 9.70054E-10 |
| *CSF1R* | 2.07738 | 4.29002E-09 | *KCNJ2* | 2.44295 | 0 | *PRDM12* | 4.1438 | 2.72216E-06 | *USP37* | 1.75253 | 4.74953E-13 |
| *CSNK1G1* | 1.45645 | 2.50748E-11 | *KCNMA1* | 2.75782 | 2.26294E-09 | *PRKCH* | 1.48232 | 9.7447E-10 | *VDR* | 1.11982 | 0.000967264 |
| *CSRNP2* | 1.83371 | 0 | *KCNQ1* | 2.29034 | 0.000630703 | *PRR15* | 2.06579 | 2.93099E-14 | *VGLL1* | 2.82282 | 0 |
| *CSRNP3* | 1.40324 | 9.53274E-07 | *KCTD1* | 1.24672 | 4.92007E-06 | *PRRG4* | 1.31476 | 1.78807E-05 | *VGLL2* | 2.84986 | 9.24029E-05 |
| *CST6* | 1.52487 | 1.68431E-07 | *KIAA0040* | 2.09517 | 0 | *PRRT4* | 2.47846 | 1.19567E-11 | *VGLL3* | 2.79602 | 0 |
| *CTHRC1* | 1.22654 | 5.33593E-08 | *KIAA0895L* | 3.25311 | 0 | *PRRX1* | 1.41244 | 1.49537E-07 | *VIPR2* | 1.1218 | 0.0024364 |
| *CTTNBP2* | 1.77368 | 1.85185E-13 | *KIAA1217* | 1.07923 | 9.86789E-07 | *PSD4* | 1.32531 | 3.08706E-06 | *VIT* | 2.87108 | 2.58869E-07 |
| *CXCL12* | 1.96457 | 3.73975E-10 | *KIAA1549L* | 3.84006 | 0 | *PTGES* | 2.33286 | 0 | *VPS29* | 1.02536 | 0.000126166 |
| *CXCR7* | 1.66991 | 7.32747E-15 | *KIAA1614* | 1.06042 | 0.00459107 | *PTHLH* | 1.66996 | 4.74286E-06 | *VPS36* | 1.31418 | 9.78261E-09 |
| *CYGB* | 2.34972 | 0 | *KIAA1671* | 1.22307 | 1.3493E-08 | *PTN* | 2.1856 | 0 | *VSNL1* | 1.54056 | 4.91253E-10 |
| *CYP24A1* | 4.55395 | 0 | *KIAA2018* | 2.04783 | 0 | *PTPLAD1* | 2.46531 | 0 | *VTCN1* | 4.08765 | 0 |
| *CYP26B1* | 1.80838 | 1.47468E-06 | *KLF12* | 1.51332 | 6.97743E-10 | *PTPLB* | 1.00682 | 4.71664E-05 | *VWA2* | 1.23564 | 7.98586E-05 |
| *CYP27C1* | 1.20164 | 2.12844E-05 | *KLF7* | 1.98197 | 0 | *PTPRE* | 1.08973 | 3.84528E-05 | *WNT1* | 4.22586 | 7.60623E-05 |
| *CYSLTR2* | 1.53755 | 2.88067E-06 | *KLK6* | 2.38868 | 0 | *PURA* | 2.42582 | 0 | *WNT10A* | 1.07735 | 0.000120742 |
| *DCAKD* | 3.1446 | 0 | *KLK7* | 1.80171 | 3.97526E-05 | *PVRL4* | 1.00768 | 0.000185914 | *WNT6* | 1.41918 | 0.000205962 |
| *DCN* | 3.76949 | 0 | *KLK8* | 2.39394 | 0.00652956 | *R3HDML* | 2.54784 | 0.00254955 | *WNT7B* | 3.17737 | 0 |
| *DCX* | 4.29515 | 0 | *KLRC2* | 2.50499 | 0.000629338 | *RAB27B* | 1.17956 | 0.0001389 | *WNT9A* | 1.59163 | 6.73281E-05 |
| *DDHD1* | 1.89867 | 4.68958E-13 | *KRT23* | 2.4522 | 6.8904E-07 | *RAB3IP* | 3.69609 | 0 | *WNT9B* | 2.06255 | 3.10423E-11 |
| *DDX60L* | 1.32216 | 0.000280826 | *KRT5* | 2.55731 | 9.99159E-09 | *RAB7A* | 2.55336 | 0 | *WWC1* | 1.79582 | 9.99201E-15 |
| *DHRS4-AS1* | 2.83192 | 0 | *L3MBTL4* | 1.72104 | 6.63762E-06 | *RAB9A* | 1.06834 | 9.10723E-05 | *WWTR1* | 1.01733 | 1.22371E-05 |
| *DLGAP4* | 1.35869 | 1.0241E-07 | *LAMC2* | 1.02441 | 1.61481E-06 | *RALGAPB* | 2.66965 | 0 | *XAGE2* | 1.96288 | 1.85237E-06 |
| *DLX1* | 1.19655 | 0.00162953 | *LAMP2* | 1.00905 | 8.39256E-06 | *RAMP1* | 2.7194 | 2.35367E-14 | *XKR4* | 2.53238 | 2.22045E-16 |
| *DLX2* | 2.1429 | 7.64842E-08 | *LARP4B* | 1.58427 | 9.34808E-14 | *RANBP17* | 1.17157 | 1.37552E-05 | *ZBED2* | 1.59251 | 3.74974E-09 |
| *DLX3* | 1.56789 | 0.000168566 | *LATS2* | 2.28012 | 0 | *RARA* | 1.13615 | 0.000250048 | *ZC3H12C* | 1.72065 | 6.21281E-13 |
| *DMRT3* | 1.81792 | 4.36984E-05 | *LAYN* | 1.18156 | 1.46142E-08 | *RASGRF1* | 1.6757 | 1.53738E-06 | *ZDHHC20* | 2.28937 | 0 |
| *DNAJC15* | 2.92525 | 0 | *LCOR* | 1.74926 | 2.23637E-08 | *RASGRP1* | 1.00876 | 0.000316045 | *ZDHHC3* | 2.0969 | 0 |
| *DNAJC6* | 3.31503 | 0 | *LDLRAD4* | 1.14759 | 0.000199585 | *RBBP9* | 2.62307 | 0 | *ZEB1* | 3.64596 | 0 |
| *DOCK1* | 2.10658 | 0 | *LEPREL1* | 2.54121 | 0 | *RBFOX1* | 1.59203 | 0.00207793 | *ZEB2* | 1.09959 | 3.89629E-05 |
| *DPYSL5* | 2.91695 | 0 | *LGALS3* | 1.06558 | 9.9472E-06 | *REEP5* | 3.44075 | 0 | *ZFHX3* | 1.08888 | 0.000262658 |
| *E2F8* | 2.77761 | 2.419E-11 | *LHFP* | 4.17914 | 0 | *RGS4* | 3.53155 | 0 | *ZFP112* | 1.12126 | 0.000285824 |
| *EDA2R* | 2.58407 | 0 | *LIMCH1* | 1.66603 | 6.31457E-11 | *RIMS2* | 1.483 | 6.75694E-07 | *ZKSCAN8* | 1.55346 | 1.11555E-12 |
| *EDNRA* | 3.1854 | 0 | *LINC00327* | 2.1131 | 3.3849E-08 | *ROR1* | 1.12905 | 2.88131E-06 | *ZNF235* | 1.06162 | 0.00187136 |
| *EFNA1* | 1.34722 | 6.71635E-10 | *LINC00346* | 1.22119 | 6.30535E-05 | *RPL23A* | 1.47952 | 3.82827E-12 | *ZNF264* | 1.03879 | 9.21704E-06 |
| *EFNB1* | 2.76845 | 0 | *LINC00475* | 2.79367 | 0.00124527 | *RPS6KB2* | 1.15888 | 1.98709E-07 | *ZNF37BP* | 1.11784 | 0.000163581 |
| *EFNB2* | 1.38252 | 3.83216E-11 | *LINC00673* | 1.04807 | 0.000467576 | *RRAGB* | 1.06335 | 6.80801E-05 | *ZNF382* | 3.04251 | 1.06581E-14 |
| *EGFR* | 1.17033 | 2.24621E-07 | *LMX1A* | 4.08335 | 1.47887E-06 | *RRP15* | 2.7169 | 0 | *ZNF449* | 1.70044 | 1.32317E-09 |
| *EHD3* | 2.4674 | 0 | *LMX1B* | 4.56137 | 1.5764E-08 | *RRP7A* | 1.83628 | 2.22045E-16 | *ZNF501* | 1.39279 | 0.00260835 |
| *EIF3IP1* | 1.11084 | 0.00122165 | *LOC100128338* | 1.47141 | 9.68419E-07 | *RUNX1* | 1.81806 | 9.43277E-09 | *ZNF503* | 1.51516 | 2.03609E-06 |
| *ELAVL2* | 1.02173 | 2.98753E-05 | *LOC100129034* | 1.51377 | 0.000180043 | *RUNX1T1* | 3.69317 | 0 | *ZNF516* | 1.21594 | 4.6442E-08 |
| *ELFN1* | 1.29362 | 4.41665E-06 | *LOC100131434* | 1.82132 | 0.00338913 | *RYR3* | 2.07191 | 1.30118E-13 | *ZNF521* | 1.72087 | 2.22045E-16 |
| *ELMOD1* | 2.97841 | 7.57417E-08 | *LOC100506394* | 1.05889 | 0.0011045 | *SAMD14* | 2.42891 | 2.26485E-13 | *ZNF618* | 2.93268 | 0 |
| *EMP2* | 1.55636 | 3.78586E-13 | *LOC100862671* | 1.33238 | 1.71204E-08 | *SBK1* | 2.95973 | 0 | *ZNF738* | 1.47657 | 5.77083E-11 |
| *EMX2* | 1.86363 | 1.05967E-05 | *LOC154761* | 1.26162 | 0.00267569 | *SCG2* | 1.55143 | 8.03739E-08 | *ZNF850* | 1.09188 | 2.56401E-05 |
| *ENPEP* | 1.64738 | 2.84217E-14 | *LOC254559* | 1.37639 | 3.02529E-05 | *SCHIP1* | 1.02831 | 0.00117667 | *ZRANB1* | 2.9657 | 0 |
| *ENPP5* | 2.11346 | 3.04001E-12 | *LOC282997* | 2.6483 | 6.23945E-14 | *SCRG1* | 2.5395 | 3.75869E-05 | *ZSWIM1* | 2.81042 | 0 |
| *EOGT* | 2.38635 | 0 | *LOC283143* | 1.47381 | 0.00144712 | *SCUBE3* | 1.13424 | 4.11505E-07 | *ZSWIM6* | 1.80407 | 2.22045E-16 |
| *EP300* | 2.11323 | 0 | *LOC344595* | 1.6749 | 2.41983E-10 | *SDK2* | 2.66059 | 0 |  |  |  |
| *EPAS1* | 1.88521 | 0 | *LOC400043* | 3.32108 | 0 | *SEMA3C* | 1.04894 | 4.97207E-07 |  |  |  |
