# Supplementary material for: Transcriptional Profiling of Ectoderm Specification to Keratinocyte Fate in Human Embryonic Stem Cells
Source: PLoS One. 2015 Apr 7;10(4):e0122493. doi: 10.1371/journal.pone.0122493 (PMC4388500; doi:10.1371/journal.pone.0122493)
Supplement: S2 Table — (DOCX) [file pone.0122493.s002.docx]

| **S2 Table. List of downregulated genes detected by transcriptional profiling of ectoderm-specified hESCs treated with DAPT versus Ethanol for 10 days.** | | | | | | | | | | | |
| --- | --- | --- | --- | --- | --- | --- | --- | --- | --- | --- | --- |
| **GENE NAME** | **FOLD CHANGE (Log2)** | **P-VALUE** | **GENE NAME** | **FOLD CHANGE (Log2)** | **P-VALUE** | **GENE NAME** | **FOLD CHANGE (Log2)** | **P-VALUE** | **GENE NAME** | **FOLD CHANGE (Log2)** | **P-VALUE** |
| *41701* | -1.52547 | 2.29E-07 | *CYP27A1* | -1.04651 | 7.43E-05 | *INHBE* | -1.99249 | 3.89E-07 | *PRSS35* | -1.29212 | 1.32E-06 |
| *AADACL3* | -3.123 | 0.000336868 | *CYP4B1* | -2.89179 | 0.000136983 | *INHBE* | -1.748498961 | 1.54E-03 | *PTGIR* | -1.45467 | 0.00042058 |
| *ABCB6* | -1.05588 | 1.01E-05 | *CYP4X1* | -2.32567 | 3.92E-11 | *INHBE* | -1.502116104 | 2.52E-03 | *PTPN5* | -1.02289 | 0.00243754 |
| *ABCC2* | -1.27934 | 0.00133954 | *DAB1* | -1.27084 | 5.35E-05 | *IQGAP2* | -1.379 | 6.57E-07 | *PTPRN* | -1.01109 | 2.87E-05 |
| *ABCG1* | -2.13011 | 0.00036802 | *DACT2* | -1.37071 | 0.000249917 | *ITLN2* | -2.95105 | 6.66E-15 | *PXK* | -1.10485 | 0.000294339 |
| *ABCG2* | -1.882889 | 0.001006883 | *DCLK1* | -1.24856 | 8.79E-06 | *KCNC4* | -1.28738 | 1.78E-05 | *RAB17* | -3.26285 | 0 |
| *ABCG3* | -1.636506 | 0.001984947 | *DENND1C* | -1.01408 | 0.0018374 | *KCNF1* | -3.20665 | 6.22E-05 | *RAB3A* | -1.17642 | 0.000341112 |
| *ABCG4* | -1.390123 | 0.002963011 | *DES* | -1.5249 | 1.93E-10 | *KCNK12* | -2.66494 | 6.51E-06 | *RAP1GAP* | -1.07032 | 0.000177246 |
| *ABHD12B* | -2.22292 | 6.69E-05 | *DGKK* | -2.75296 | 0 | *KCNK5* | -1.35342 | 8.52E-08 | *RASD1* | -1.28106 | 0.00105971 |
| *ACE* | -2.24017 | 2.20E-08 | *DIO2* | -2.09116 | 3.36E-09 | *KCNS1* | -1.11922 | 4.82E-05 | *RASSF10* | -1.03126 | 0.0024792 |
| *ACE2* | -2.3305 | 2.98E-08 | *DIO3* | -3.12265 | 0 | *KCNS3* | -1.0055 | 0.000273069 | *RASSF6* | -2.1797 | 4.69E-07 |
| *ACP5* | -1.26602 | 0.000454803 | *DIO3* | -1.842572416 | 1.17E-03 | *KCNV1* | -2.88267 | 2.44E-13 | *RBFOX3* | -1.09535 | 0.00661963 |
| *ACTG2* | -1.13669 | 0.000639215 | *DIO4* | -1.596189558 | 2.14E-03 | *KDR* | -1.3584 | 2.04E-10 | *RBP4* | -2.28758 | 2.66E-07 |
| *ACVRL1* | -1.50984 | 2.31E-05 | *DIO5* | -1.349806701 | 3.12E-03 | *KEL* | -2.21881 | 3.09E-12 | *RBPMS2* | -1.24524 | 6.27E-08 |
| *ACY1* | -1.215 | 2.59E-05 | *DKFZP686I15217* | -1.27718 | 0.00425594 | *KIAA1244* | -1.12392 | 9.82E-06 | *RENBP* | -1.21529 | 0.000237387 |
| *ADAMTS10* | -1.417002 | 0.002856313 | *DKK4* | -2.95651 | 5.12E-08 | *KIF12* | -1.20896 | 0.00354945 | *RGCC* | -1.01961 | 0.00283769 |
| *ADAMTS8* | -1.92899 | 0.000172502 | *DLGAP3* | -1.3818 | 0.000471822 | *KIF1A* | -1.1622 | 8.72E-08 | *RGN* | -1.96239 | 0.00640816 |
| *ADAMTS9* | -1.663384 | 0.001878249 | *DLK1* | -1.37662 | 1.75E-10 | *KLHL6* | -1.275 | 0.000151468 | *RGN* | -1.726100 | 0.001629288 |
| *ADAMTSL2* | -1.40343 | 1.35E-06 | *DNAJC22* | -2.47271 | 1.29E-06 | *KLKB1* | -1.36748 | 4.75E-06 | *RGN* | -1.479717662 | 0.002607351 |
| *ADAP1* | -1.22258 | 0.000798667 | *DNMT3B* | -1.02603 | 5.64E-06 | *KLRG2* | -2.4252 | 6.67E-05 | *RHAG* | -1.43327 | 6.47E-06 |
| *ADCK3* | -1.25477 | 9.30E-07 | *DPEP1* | -1.94425 | 4.93E-06 | *KYNU* | -1.93793 | 0.00103981 | *RHEBL1* | -1.12942 | 0.00557223 |
| *ADCY2* | -2.76904 | 0 | *DPEP2* | -1.708181766 | 1.70E-03 | *KYNU* | -1.690263013 | 0.001771551 | *RIMKLA* | -1.43835 | 4.36E-07 |
| *ADCY8* | -2.21716 | 4.02E-11 | *DPEP3* | -1.461798909 | 2.68E-03 | *KYNU* | -1.443880156 | 0.002749615 | *RNASE1* | -1.9263 | 3.93E-09 |
| *ADD2* | -1.3788 | 1.02E-07 | *DPEP3* | -1.2627 | 8.67E-05 | *LAPTM4B* | -1.04719 | 1.05E-06 | *RNASE2* | -1.658905 | 1.90E-03 |
| *ADM* | -1.5964 | 8.85E-12 | *DPP4* | -2.78947 | 0 | *LARP7* | -1.06874 | 1.75E-05 | *RNASE3* | -1.412522 | 2.87E-03 |
| *ADM2* | -1.34547 | 6.46E-07 | *DPPA2* | -2.49185 | 2.93E-12 | *LCN10* | -2.22155 | 2.23E-05 | *RPP25* | -1.00022 | 0.000584731 |
| *ADORA1* | -1.15418 | 6.01E-05 | *DPPA3* | -2.82333 | 8.79E-07 | *LCN15* | -3.11651 | 0 | *RSPO2* | -1.12647 | 2.52E-05 |
| *AFP* | -2.76824 | 0 | *DPPA4* | -1.07801 | 2.23E-07 | *LDB2* | -1.56307 | 1.55E-07 | *RTP1* | -1.28187 | 0.00675375 |
| *AGR2* | -2.61174 | 0.00608406 | *DTWD2* | -1.5543 | 9.15E-08 | *LECT1* | -1.12161 | 4.53E-06 | *RYR2* | -1.15055 | 0.000100393 |
| *AIM1* | -1.54499 | 4.76E-07 | *DUSP6* | -1.10935 | 1.94E-07 | *LEFTY1* | -2.19495 | 1.61E-08 | *S100A14* | -3.28748 | 0 |
| *AKR1B10* | -2.66269 | 9.90E-07 | *DUSP9* | -1.34898 | 0.000267314 | *LGALS1* | -1.6029 | 5.93E-13 | *S100Z* | -2.92402 | 4.43E-06 |
| *AKR1B15* | -2.56965 | 0.00138337 | *DYNC1I1* | -1.16349 | 0.000191987 | *LGALS2* | -2.04813 | 0.00014068 | *SBSPON* | -1.32228 | 7.00E-06 |
| *AKR1C1* | -1.13887 | 0.000770293 | *ECM1* | -1.51939 | 2.44E-08 | *LGALS3* | -1.793295844 | 0.001362543 | *SCNN1A* | -1.52542 | 5.58E-08 |
| *AKR1C3* | -1.39737 | 0.000481591 | *ECSCR* | -1.33535 | 0.000225779 | *LGALS4* | -1.546912987 | 0.002340607 | *SEMG1* | -2.61917 | 0.0043669 |
| *AKR1D1* | -2.12741 | 0.000415291 | *EGF* | -1.20211 | 0.000687422 | *LGI2* | -1.0916 | 8.67E-05 | *SEPHS1* | -1.17993 | 6.73E-07 |
| *AKR1D2* | -1.873930 | 0.001042449 | *EGFL7* | -1.36917 | 1.18E-06 | *LHX1* | -3.49764 | 0 | *SEPP1* | -1.0538 | 1.43E-05 |
| *AKR1D3* | -1.627547 | 0.002020513 | *EGFLAM* | -1.49183 | 3.77E-07 | *LHX8* | -1.55906 | 0.00348849 | *SERPINA1* | -2.63312 | 8.81E-13 |
| *AKR1D4* | -1.381164 | 0.002998577 | *ELMO1* | -1.93402 | 2.91E-06 | *LINC-ROR* | -2.21606 | 1.24E-07 | *SERPINB10* | -1.757458 | 0.001504807 |
| *ALDH1A1* | -1.12653 | 0.000689687 | *ELMO2* | -1.685783325 | 1.79E-03 | *LINC00261* | -2.86982 | 0 | *SERPINB11* | -1.511075 | 0.002482871 |
| *ALDOC* | -1.01202 | 3.53E-06 | *ELMO3* | -1.439400468 | 2.77E-03 | *LINC00479* | -3.45582 | 5.13E-10 | *SERPINB9* | -2.00076 | 0 |
| *ALPL* | -1.5935 | 2.09E-11 | *ENHO* | -1.18156 | 0.000799234 | *LINC00617* | -2.2975 | 0 | *SERPINE2* | -1.35907 | 1.18E-08 |
| *AMBP* | -2.20962 | 0.000192538 | *ENPP1* | -1.42895 | 8.11E-09 | *LINC00698* | -1.13211 | 0.0036125 | *SEZ6L2* | -1.11645 | 2.52E-05 |
| *AMN* | -3.57017 | 0.00623884 | *ENTPD2* | -2.28783 | 5.01E-05 | *LOC100130899* | -2.27415 | 1.51E-13 | *SFRP5* | -2.79936 | 1.28E-07 |
| *ANKS4B* | -3.23458 | 3.63E-09 | *EOMES* | -3.52484 | 0 | *LOC100287314* | -1.38672 | 0.00038937 | *SH3TC1* | -1.39706 | 6.43E-05 |
| *ANO1* | -1.22326 | 0.00308381 | *EPHA6* | -1.55336 | 0.000156695 | *LOC100506776* | -2.43446 | 3.98E-12 | *SHH* | -2.067 | 4.56E-05 |
| *ANPEP* | -1.07471 | 0.000293455 | *ESPN* | -1.24109 | 0.0024094 | *LOC100506990* | -1.01625 | 0.000434792 | *SHH* | -1.824653 | 1.24E-03 |
| *AOC3* | -1.22142 | 0.000673841 | *ESRG* | -1.98362 | 0 | *LOC147646* | -1.93068 | 0.00118236 | *SHH* | -1.578270 | 2.22E-03 |
| *AOX1* | -1.02206 | 0.00307826 | *ESRG* | -1.744019273 | 0.001558156 | *LOC147647* | -1.681303636 | 0.001807117 | *SHH* | -1.331887 | 3.19E-03 |
| *AP3B2* | -1.44582 | 4.42E-05 | *ESRG* | -1.497636416 | 0.002536219 | *LOC147648* | -1.434920779 | 0.002785181 | *SHISA6* | -2.04918 | 2.66E-11 |
| *APOA1* | -4.02426 | 0 | *ETV4* | -1.3991 | 5.98E-10 | *LOC151009* | -2.10117 | 1.75E-06 | *SHISA7* | -1.797775 | 1.34E-03 |
| *APOA2* | -4.30625 | 0 | *EVX1* | -2.94038 | 2.71E-09 | *LOC151010* | -1.851531792 | 1.13E-03 | *SHISA8* | -1.551392 | 2.32E-03 |
| *APOA4* | -4.67882 | 0 | *EXOC3L2* | -1.42413 | 0.00072802 | *LOC151011* | -1.605148935 | 2.11E-03 | *SHOX2* | -2.58804 | 2.71E-06 |
| *APOB* | -3.93668 | 0 | *EXOSC5* | -1.08672 | 0.00017652 | *LOC151012* | -1.358766078 | 3.09E-03 | *SLC13A4* | -1.40466 | 0.00045706 |
| *APOBEC3D* | -1.20232 | 7.48E-05 | *F10* | -2.1575 | 7.36E-13 | *LOC440925* | -2.53696 | 8.44E-06 | *SLC13A5* | -1.43934 | 0.00105046 |
| *APOC1* | -2.52435 | 0 | *F2* | -3.35697 | 6.26E-13 | *LOC650368* | -1.16789 | 3.79E-05 | *SLC16A10* | -1.48452 | 3.33E-05 |
| *APOC1P1* | -2.86913 | 4.39E-10 | *FABP1* | -2.76538 | 7.30E-06 | *LPAR3* | -1.36875 | 0.000524819 | *SLC17A9* | -1.05445 | 3.36E-05 |
| *APOC3* | -3.85825 | 0.000921184 | *FADS6* | -2.52853 | 2.21E-05 | *LRAT* | -1.31397 | 5.43E-06 | *SLC1A7* | -3.98249 | 0.000133232 |
| *AQP10* | -3.58634 | 0.000570212 | *FAM124A* | -1.01823 | 0.000195286 | *LRP8* | -1.18487 | 8.80E-07 | *SLC22A2* | -3.07198 | 6.63E-05 |
| *AQP4* | -2.29786 | 0.00212789 | *FAM124B* | -2.3553 | 3.24E-07 | *LRRC16B* | -1.294 | 0.00189882 | *SLC25A18* | 2.76538 | 3.30E-10 |
| *AREG* | -2.21007 | 8.10E-05 | *FAM155A* | -1.09204 | 0.000653225 | *LRRC32* | -1.52856 | 1.58E-11 | *SLC27A3* | -1.36956 | 7.29E-07 |
| *ARL4D* | -1.32804 | 1.04E-07 | *FAM155B* | -2.05594 | 1.56E-12 | *MAGEA4* | -1.3727 | 0.00636 | *SLC29A1* | -1.14989 | 8.35E-06 |
| *ART4* | -1.42404 | 5.55E-07 | *FAM155B* | -1.806734909 | 1.31E-03 | *MAL2* | -1.15995 | 1.28E-06 | *SLC39A4* | -2.45851 | 3.23E-06 |
| *ART5* | -1.35315 | 0.00419969 | *FAM155B* | -1.560352052 | 2.29E-03 | *MANEAL* | -1.31403 | 0.00642353 | *SLC43A1* | -1.57321 | 7.46E-07 |
| *ASGR2* | -3.37622 | 0.000325043 | *FAM163B* | -1.6061 | 4.25E-05 | *MASP1* | -2.11619 | 1.10E-12 | *SLC44A4* | -2.29465 | 2.05E-07 |
| *ASPHD1* | -1.52249 | 2.41E-05 | *FAM174B* | -1.0371 | 0.000152949 | *MASP2* | -1.864970857 | 1.08E-03 | *SLC51A* | -1.43386 | 0.00155273 |
| *ASRGL1* | -1.341 | 6.57E-07 | *FAM184A* | -2.38538 | 7.73E-11 | *MASP3* | -1.618588 | 2.06E-03 | *SLC5A9* | -3.87189 | 4.06E-11 |
| *ASS1* | -1.04509 | 8.76E-06 | *FAM189A2* | -1.07821 | 0.000340028 | *MASP4* | -1.372205143 | 3.03E-03 | *SLC6A17* | -2.39814 | 1.04E-12 |
| *ATCAY* | -1.01903 | 8.25E-05 | *FAM195A* | -1.24591 | 0.00123624 | *MAT1A* | -1.59907 | 0.0019837 | *SLC6A19* | -3.12879 | 1.03E-09 |
| *ATP12A* | -2.08127 | 1.33E-09 | *FAM19A5* | -1.09376 | 0.000261546 | *MIXL1* | -1.42254 | 0.00393687 | *SLCO4A1* | -1.97379 | 6.90E-07 |
| *ATP12A* | -1.838092 | 1.18E-03 | *FAM46C* | -1.0729 | 0.00130698 | *MLXIPL* | -1.49757 | 0.00171933 | *SLCO4A2* | -1.735059 | 1.59E-03 |
| *ATP12A* | -1.591709 | 2.16E-03 | *FAM65B* | -1.25952 | 0.000128749 | *MMP1* | -2.04563 | 7.73E-12 | *SLCO4A3* | -1.488677 | 2.57E-03 |
| *ATP12A* | -1.345327 | 3.14E-03 | *FAM71F1* | -1.21063 | 0.000296836 | *MMP2* | -1.784336468 | 1.40E-03 | *SLCO5A1* | -1.41379 | 0.00113593 |
| *ATP1A3* | -1.51248 | 4.11E-07 | *FAM83F* | -2.20136 | 1.16E-10 | *MMP25* | -1.3809 | 9.42E-05 | *SLITRK2* | -2.48537 | 0.000356675 |
| *ATP2A3* | -1.32106 | 1.04E-05 | *FBLN2* | -2.31024 | 0 | *MMP3* | -1.53795361 | 2.38E-03 | *SLITRK4* | -1.2134 | 0.00010758 |
| *B3GALT1* | -1.01633 | 0.00380234 | *FBXL16* | -1.07947 | 0.00366291 | *MNX1* | -3.56941 | 8.21E-07 | *SNAI3* | -1.46846 | 0.00450343 |
| *B4GALNT3* | -1.16562 | 1.50E-05 | *FDXR* | -1.54553 | 6.58E-07 | *MOCOS* | -1.52097 | 0.000338716 | *SNCB* | -1.37788 | 0.0037867 |
| *BAIAP2L2* | -1.40407 | 0.000949027 | *FGA* | -2.8226 | 7.44E-05 | *MPP1* | -1.30161 | 4.57E-06 | *SOAT2* | -3.05684 | 6.11E-14 |
| *BBS9* | -1.13183 | 1.11E-05 | *FGB* | -1.955 | 1.49E-08 | *MRGPRF* | -1.06185 | 0.000407463 | *SOCS1* | -1.37202 | 7.71E-05 |
| *BEND4* | -1.16283 | 0.000564739 | *FGB* | -1.717141143 | 1.66E-03 | *MRM1* | -1.12183 | 0.000484746 | *SOCS2* | -1.2189 | 5.48E-05 |
| *BEST2* | -2.5956 | 0.000359321 | *FGB* | -1.470758286 | 2.64E-03 | *MT1E* | -2.49019 | 1.13E-13 | *SOD3* | -1.11472 | 0.00127131 |
| *BEX1* | -1.01727 | 3.84E-05 | *FGF12* | -1.1609 | 0.000161877 | *MT1F* | -2.64071 | 1.56E-10 | *SOX17* | -3.2151 | 2.22E-16 |
| *BEX5* | -1.4901 | 3.57E-05 | *FGF17* | -3.3749 | 1.00E-11 | *MT1G* | -2.88843 | 0 | *SOX3* | -1.02229 | 0.00107799 |
| *BGN* | -1.13618 | 7.82E-07 | *FGF19* | -1.36276 | 2.22E-05 | *MT1H* | -2.95668 | 0 | *SP5* | -2.14742 | 4.65E-12 |
| *BHLHE22* | -2.42745 | 0.000323003 | *FGFR3* | -1.37045 | 4.34E-08 | *MT1P2* | -2.88842 | 0.000703233 | *SP6* | -1.900808 | 9.36E-04 |
| *BMP10* | -2.12468 | 3.14E-12 | *FGFR4* | -2.1283 | 2.00E-15 | *MT1X* | -2.58351 | 0 | *SP7* | -1.654425 | 1.91E-03 |
| *BMP11* | -1.869450 | 1.06E-03 | *FGFR5* | -1.878409922 | 1.02E-03 | *MT2A* | -1.05735 | 0.000118497 | *SP8* | -1.408042 | 2.89E-03 |
| *BMP12* | -1.623067 | 2.04E-03 | *FGFR6* | -1.632027065 | 2.00E-03 | *MTTP* | -3.61541 | 0 | *SPARCL1* | -1.22581 | 0.00383375 |
| *BMP13* | -1.376684 | 3.02E-03 | *FGFR7* | -1.385644208 | 2.98E-03 | *MYBPC2* | -1.56343 | 0.000203471 | *SPATA13* | -1.32071 | 2.47E-05 |
| *BMP2* | -1.09471 | 2.52E-06 | *FLRT3* | -1.07489 | 4.40E-05 | *MYCT1* | -1.11152 | 0.000935168 | *SPATA16* | -2.90866 | 9.90E-05 |
| *BRSK2* | -1.39454 | 0.000202569 | *FLVCR1-AS1* | -1.53882 | 1.85E-06 | *MYEOV* | -2.01034 | 0.000464676 | *SPON2* | -1.4049 | 0.000151976 |
| *BSPRY* | -1.16241 | 0.00064106 | *FOLR1* | -1.25496 | 0.000399197 | *MYEOV* | -1.770897403 | 0.001451458 | *SRPX2* | -1.2907 | 4.92E-08 |
| *BTK* | -1.59324 | 0.00178289 | *FOSL1* | -1.35075 | 2.47E-07 | *MYEOV* | -1.524514545 | 0.002429522 | *SRRM3* | -1.04261 | 0.00142973 |
| *C16orf74* | -1.2019 | 0.00564672 | *FOXA1* | -2.06148 | 6.58E-07 | *MYH2* | -3.10674 | 2.40E-05 | *SRY* | -1.26027 | 0.00189656 |
| *C1orf61* | -2.44744 | 5.01E-06 | *FOXA2* | -3.69424 | 0 | *MYL3* | -2.50506 | 4.24E-11 | *SSTR1* | -2.37697 | 0.000131111 |
| *C1orf64* | -1.97573 | 0.00094557 | *FOXA2* | -1.815694286 | 1.27E-03 | *MYL4* | -1.55363 | 1.90E-06 | *SSTR2* | -1.16663 | 4.55E-05 |
| *C1orf65* | -1.739539 | 0.001575939 | *FOXA3* | -1.569311429 | 2.25E-03 | *MYO18B* | -2.1539 | 1.13E-07 | *ST8SIA4* | -2.65452 | 1.37E-06 |
| *C1orf66* | -1.493156 | 0.002554002 | *FOXA4* | -1.322928571 | 3.23E-03 | *MYO3A* | -1.25315 | 0.000243299 | *STC1* | -1.12678 | 9.42E-07 |
| *C21orf88* | -1.34258 | 8.90E-05 | *FOXD1* | -1.51101 | 4.70E-06 | *MYOZ2* | -2.3686 | 1.65E-09 | *STEAP1* | -1.08518 | 0.00449896 |
| *C2orf72* | -1.26879 | 3.15E-05 | *FOXF1* | -2.00942 | 8.16E-11 | *N4BP3* | -1.082 | 1.57E-05 | *STEAP1B* | -1.45801 | 0.00151838 |
| *C2orf81* | -1.18901 | 3.14E-05 | *FOXF2* | -1.766417714 | 1.47E-03 | *NAALAD2* | -1.4091 | 3.27E-08 | *STEAP3* | -1.17265 | 0.000395358 |
| *C3* | -1.39182 | 2.73E-06 | *FOXF3* | -1.520034857 | 2.45E-03 | *NANOG* | -2.35596 | 2.22E-16 | *SUSD2* | -1.46836 | 1.37E-05 |
| *CA12* | -1.01101 | 0.000800581 | *FOXJ1* | -2.86234 | 0.000161071 | *NAT8L* | -1.15405 | 0.000388777 | *SUSD3* | -2.37294 | 8.21E-07 |
| *CA2* | -1.52148 | 3.91E-11 | *FOXQ1* | -3.90912 | 0 | *NCR1* | -3.11102 | 0.00547849 | *SYNPO2L* | -2.10658 | 4.31E-12 |
| *CA4* | -2.06817 | 3.69E-08 | *FRY* | -1.21608 | 2.48E-05 | *NID2* | -1.20235 | 1.17E-08 | *SYNPO2L* | -1.856011 | 1.11E-03 |
| *CA5* | -1.829133 | 1.22E-03 | *FRZB* | -1.32318 | 2.37E-07 | *NKD2* | -1.5221 | 0.00138694 | *SYNPO2L* | -1.609628 | 2.09E-03 |
| *CA6* | -1.582750 | 2.20E-03 | *FSHR* | -2.55804 | 1.38E-11 | *NKX1-2* | -1.93056 | 0.00158593 | *SYNPO2L* | -1.363245 | 3.07E-03 |
| *CA7* | -1.336367 | 3.18E-03 | *FZD4* | -1.12869 | 5.83E-07 | *NKX1-3* | -1.676823948 | 0.0018249 | *SYT12* | -1.514 | 0.00292401 |
| *CACNA1E* | -2.7189 | 9.84E-07 | *G0S2* | -1.93828 | 0.00538489 | *NKX1-4* | -1.430441091 | 0.002802964 | *SYT6* | -1.17795 | 0.000194891 |
| *CADPS* | -1.93832 | 1.72E-06 | *G0S3* | -1.694742701 | 0.001753768 | *NMRK2* | -2.56787 | 0 | *SYTL4* | -1.01254 | 0.00192965 |
| *CADPS* | -1.699222 | 1.74E-03 | *G0S4* | -1.448359844 | 0.002731832 | *NODAL* | -2.23333 | 2.89E-15 | *SYTL5* | -1.40161 | 1.42E-09 |
| *CADPS* | -1.452839 | 2.71E-03 | *GABRE* | -1.59209 | 0.000809439 | *NPAS1* | -1.24366 | 0.00290282 | *T* | -2.0611 | 5.54E-07 |
| *CALB1* | -2.00565 | 8.04E-08 | *GABRQ* | -1.40668 | 0.000367143 | *NPNT* | -1.08061 | 5.86E-06 | *T* | -1.811214 | 1.29E-03 |
| *CALB2* | -1.761938 | 1.49E-03 | *GAD1* | -1.20435 | 0.000270236 | *NPR1* | -1.46042 | 2.48E-07 | *T* | -1.56483 | 2.27E-03 |
| *CALB3* | -1.515555 | 2.47E-03 | *GAL* | -3.38365 | 0 | *NPTXR* | -1.55289 | 2.43E-08 | *TCEA3* | -1.3911 | 1.77E-05 |
| *CAMKV* | -1.00885 | 0.000189416 | *GALNT14* | -1.13348 | 0.00099491 | *NTS* | -2.99834 | 0 | *TCL1B* | -3.30455 | 6.36E-07 |
| *CAPN13* | -1.94612 | 0.000142222 | *GATA2* | -1.27588 | 1.11E-05 | *NUDT15* | -1.01065 | 7.15E-06 | *TCN2* | -1.17701 | 3.26E-06 |
| *CAPN14* | -1.712661 | 0.001682637 | *GATA4* | -2.37373 | 0 | *OCEL1* | -1.00749 | 0.000712656 | *TDGF1* | -2.2075 | 0 |
| *CAPN15* | -1.466278 | 0.0026607 | *GATA6* | -1.60079 | 9.23E-10 | *ONECUT1* | -2.30747 | 0.00020205 | *TFF1* | -4.95527 | 1.93E-06 |
| *CBLC* | -1.39314 | 0.000133654 | *GATM* | -3.2423 | 0 | *ONECUT2* | -2.21095 | 5.33E-10 | *TGFBR3* | -1.56919 | 6.07E-10 |
| *CBR1* | -1.1458 | 3.07E-06 | *GBP4* | -1.28477 | 3.37E-06 | *OPRK1* | -2.17929 | 1.08E-05 | *THY1* | -1.30097 | 8.79E-10 |
| *CBS* | -1.00509 | 0.000261669 | *GCHFR* | -1.28883 | 3.14E-05 | *OR52A1* | -3.75321 | 0.00123169 | *TLE2* | -2.52589 | 5.72E-09 |
| *CBX7* | -1.21299 | 2.75E-05 | *GDF15* | -2.50418 | 0 | *ORAI1* | -1.13551 | 4.82E-05 | *TLR8-AS1* | -2.57598 | 0.000992577 |
| *CCBL1* | -1.10245 | 1.54E-05 | *GDF2* | -2.49642 | 2.59E-08 | *OSBP2* | -1.24807 | 0.000618925 | *TM4SF1* | -1.10885 | 5.99E-07 |
| *CCDC129* | -3.63944 | 1.45E-07 | *GDF3* | -2.76482 | 0 | *OVGP1* | -1.01081 | 0.00126598 | *TM4SF18* | -1.4824 | 0.00419155 |
| *CCDC141* | -3.07879 | 1.55E-15 | *GEM* | -1.09523 | 0.00138724 | *OXLD1* | -1.27168 | 0.000312549 | *TMEM132D* | -1.43809 | 3.77E-06 |
| *CCDC151* | -1.49419 | 0.000280689 | *GFPT2* | -1.49324 | 8.13E-12 | *PABPC4L* | -1.18503 | 1.70E-06 | *TMEM249* | -2.7011 | 0.00425494 |
| *CCDC81* | -1.54129 | 1.37E-05 | *GGT1* | -1.40851 | 0.00378819 | *PACSIN1* | -1.53244 | 4.18E-06 | *TMEM27* | -1.08324 | 0.000473367 |
| *CCKBR* | -2.32764 | 0 | *GJA4* | -2.59036 | 2.22E-16 | *PADI1* | -1.41759 | 0.00230563 | *TMEM37* | -1.05695 | 0.000521011 |
| *CCL26* | -2.16602 | 0.000303078 | *GJA5* | -2.01328 | 8.79E-11 | *PADI3* | -1.55413 | 0.000436907 | *TMEM38A* | -1.47224 | 8.43E-05 |
| *CCM2L* | -1.31285 | 0.00112188 | *GJA6* | -1.775377091 | 1.43E-03 | *PALD1* | -1.28262 | 1.13E-06 | *TMEM52* | -1.35364 | 0.00168459 |
| *CD177* | -3.9536 | 0 | *GJA7* | -1.528994234 | 2.41E-03 | *PAPSS2* | -1.00093 | 2.38E-05 | *TMEM59L* | -1.41709 | 0.000164966 |
| *CD1D* | -1.28974 | 0.00159404 | *GLYATL2* | -2.04677 | 0.00548498 | *PARD6A* | -1.47831 | 0.000899106 | *TMPRSS11E* | -3.17997 | 2.22E-16 |
| *CD200* | -1.01693 | 0.000302976 | *GLYATL3* | -1.788816156 | 0.001380326 | *PARVB* | -1.15902 | 1.71E-05 | *TMPRSS2* | -2.24924 | 1.69E-13 |
| *CD300E* | -1.45766 | 0.00108219 | *GLYATL4* | -1.542433299 | 0.00235839 | *PAX2* | -2.06348 | 0.000246257 | *TNFRSF11A* | -2.36351 | 1.33E-11 |
| *CD70* | -1.43775 | 0.00122982 | *GMPR* | -1.49302 | 4.42E-06 | *PAX3* | -1.820173974 | 0.001255845 | *TNFSF10* | -1.667864 | 1.86E-03 |
| *CDKN1C* | -1.20854 | 9.92E-06 | *GNRH2* | -3.0591 | 0 | *PAX4* | -1.573791117 | 0.002233909 | *TNFSF11* | -1.421481 | 2.84E-03 |
| *CER1* | -2.01867 | 2.11E-11 | *GPD1* | -2.83809 | 1.41E-06 | *PAX5* | -1.32740826 | 0.003211973 | *TNFSF9* | -1.92909 | 2.32E-08 |
| *CER2* | -1.779856 | 1.42E-03 | *GPR124* | -1.15664 | 4.57E-07 | *PCDH20* | -2.13048 | 2.30E-08 | *TNNC1* | -1.08335 | 6.54E-06 |
| *CER3* | -1.533473 | 2.39E-03 | *GPR128* | -3.54288 | 0.000352526 | *PCDH21* | -1.887369299 | 9.89E-04 | *TPK1* | -1.08695 | 0.00416164 |
| *CHAC1* | -1.53385 | 3.11E-07 | *GPR160* | -1.29274 | 3.57E-05 | *PCDH22* | -1.640986442 | 1.97E-03 | *TPPP3* | -1.43027 | 1.80E-05 |
| *CHCHD10* | -1.25801 | 1.57E-05 | *GPR176* | -1.12463 | 8.39E-06 | *PCDH23* | -1.394603584 | 2.95E-03 | *TPST2* | -1.11774 | 2.70E-06 |
| *CHDH* | -1.07443 | 0.000720627 | *GPR3* | -1.03998 | 0.00111661 | *PCED1B* | -1.02479 | 0.00185626 | *TRIM4* | -3.52253 | 3.71E-06 |
| *CHRM3* | -1.12436 | 0.00398546 | *GPR55* | -2.34965 | 9.16E-07 | *PCK2* | -1.12385 | 7.58E-06 | *TRPA1* | -3.6491 | 3.02E-10 |
| *CHST13* | -2.5032 | 0.00354025 | *GRB7* | -1.18544 | 0.000564017 | *PCSK9* | -1.23531 | 1.13E-05 | *TRPM2* | -2.1425 | 9.60E-08 |
| *CHST4* | -1.41515 | 0.00487316 | *GREM2* | -1.48297 | 0.000522185 | *PDE2A* | -1.31391 | 4.79E-06 | *TRPM3* | -1.896328 | 9.54E-04 |
| *CHST8* | -2.2174 | 2.42E-09 | *GRID2* | -1.94335 | 8.36E-13 | *PDE4A* | -1.04796 | 0.00496052 | *TRPM4* | -1.649945 | 1.93E-03 |
| *CILP2* | -1.43717 | 7.72E-05 | *GRID3* | -1.703702078 | 1.72E-03 | *PDE4B* | -1.30302 | 3.22E-05 | *TRPM5* | -1.403562 | 2.91E-03 |
| *CKB* | -2.51193 | 0 | *GRID4* | -1.457319221 | 2.70E-03 | *PDGFRA* | -1.48423 | 9.49E-10 | *TRPV2* | -1.56012 | 6.10E-09 |
| *CKM* | -3.43162 | 0.000262825 | *GRPR* | -2.22068 | 0 | *PDZD4* | -2.21591 | 0 | *TSPAN33* | -1.282 | 9.36E-07 |
| *CLDN11* | -2.52921 | 6.66E-16 | *GSC* | -2.90272 | 1.37E-05 | *PDZK1* | -1.44376 | 0.00406405 | *TTR* | -3.61572 | 0 |
| *CLDN19* | -1.99427 | 1.17E-07 | *GYLTL1B* | -1.41666 | 7.16E-07 | *PDZK1P1* | -1.48706 | 0.000422504 | *TUBB2A* | -1.04168 | 1.44E-06 |
| *CLDN20* | -1.752978 | 1.52E-03 | *HABP2* | -2.76679 | 1.90E-05 | *PENK* | -2.23305 | 5.04E-14 | *TUBB2B* | -1.08068 | 9.91E-07 |
| *CLDN21* | -1.506595 | 2.50E-03 | *HAGHL* | -1.32112 | 0.000463489 | *PGF* | -1.10442 | 0.00222754 | *TUBB4A* | -1.05755 | 1.35E-05 |
| *CLEC1B* | -2.1123 | 1.03E-08 | *HAS2* | -1.51116 | 2.31E-11 | *PGM1* | -1.09387 | 5.86E-06 | *TYRP1* | -1.19993 | 0.00373063 |
| *CLEC1B* | -1.860491 | 1.10E-03 | *HAVCR1* | -2.9964 | 3.83E-07 | *PHC1* | -1.14089 | 7.71E-05 | *UAP1L1* | -1.31476 | 2.21E-05 |
| *CLEC1B* | -1.614108 | 2.07E-03 | *HCN2* | -1.54619 | 3.47E-05 | *PHGDH* | -1.00634 | 1.45E-06 | *UCMA* | -2.81665 | 0.00639159 |
| *CLEC1B* | -1.367725 | 3.05E-03 | *HCP5* | -1.30806 | 0.000147235 | *PHYHIP* | -1.29158 | 0.00108029 | *UGT2B11* | -2.13162 | 3.87E-05 |
| *CLEC2B* | -3.15568 | 1.05E-06 | *HERC5* | -1.12496 | 5.36E-05 | *PIF1* | -1.28353 | 1.81E-06 | *UGT2B12* | -1.891848 | 9.71E-04 |
| *CLMP* | -1.03344 | 5.93E-05 | *HEY2* | -1.17917 | 2.08E-05 | *PIK3R5* | -1.39915 | 1.25E-05 | *UGT2B13* | -1.645463 | 1.95E-03 |
| *CMTM8* | -1.11099 | 0.0038553 | *HHEX* | -1.55811 | 0.000817709 | *PIM2* | -1.47387 | 4.76E-10 | *UGT2B14* | -1.399083 | 2.93E-03 |
| *CNIH3* | -1.35266 | 7.76E-06 | *HLA-DQB1* | -1.10607 | 0.000261706 | *PIPOX* | -1.4948 | 2.96E-07 | *UNC13A* | -1.14248 | 6.58E-06 |
| *CNNM2* | -1.2323 | 1.58E-05 | *HLA-DRB5* | -2.31024 | 0.0028692 | *PITX2* | -1.11796 | 1.26E-05 | *UNC93A* | -4.25981 | 0.000238897 |
| *COBL* | -1.14654 | 1.06E-06 | *HMOX1* | -2.10042 | 5.06E-14 | *PLA2G12B* | -2.55823 | 1.81E-05 | *UPK1A* | -1.16464 | 0.00262564 |
| *COCH* | -2.07696 | 3.85E-13 | *HMOX2* | -1.847052104 | 1.15E-03 | *PLA2G16* | -1.15076 | 7.94E-05 | *USH1C* | -2.31141 | 0.00140932 |
| *COCH* | -1.833613 | 1.20E-03 | *HMOX3* | -1.600669247 | 2.13E-03 | *PLA2G3* | -1.1137 | 0.000511394 | *USP44* | -1.30731 | 7.05E-06 |
| *COCH* | -1.587230 | 2.18E-03 | *HMOX4* | -1.35428639 | 3.11E-03 | *PLLP* | -1.276 | 0.000795046 | *VCAN* | -1.30822 | 8.49E-08 |
| *COCH* | -1.340847 | 3.16E-03 | *HNF1B* | -1.41599 | 0.000246853 | *PLXNB3* | -1.5746 | 0.00015249 | *VENTX* | -2.24824 | 4.26E-11 |
| *COL9A2* | -1.96714 | 6.15E-14 | *HNF4A* | -3.69908 | 8.66E-15 | *PODN* | -1.32205 | 0.000828618 | *VIL1* | -2.80326 | 0 |
| *COL9A3* | -1.730580 | 1.61E-03 | *HP* | -4.62936 | 1.14E-11 | *POLR3G* | -1.19336 | 1.24E-06 | *VRTN* | -1.51264 | 1.42E-10 |
| *COL9A4* | -1.484197 | 2.59E-03 | *HPGD* | -1.23094 | 1.98E-05 | *POU5F1* | -2.34451 | 0 | *VTN* | -2.59634 | 0 |
| *COLEC11* | -2.05041 | 8.35E-07 | *HPN* | -1.24089 | 0.000105027 | *POU5F1B* | -2.15299 | 0 | *VWDE* | -1.23752 | 1.35E-05 |
| *COLEC12* | -1.802255 | 1.33E-03 | *HPR* | -4.57818 | 5.83E-07 | *POU5F1P3* | -2.3991 | 0 | *WBSCR17* | -1.3151 | 4.44E-08 |
| *COLEC13* | -1.555872 | 2.31E-03 | *HRASLS5* | -1.52043 | 2.28E-05 | *POU5F1P4* | -2.199 | 7.77E-06 | *WNT11* | -1.02449 | 0.00102476 |
| *CPA1* | -1.52479 | 4.02E-05 | *HRC* | -1.02387 | 0.000320069 | *PPAPDC3* | -1.21634 | 0.00658029 | *YBX2* | -1.13655 | 0.000603434 |
| *CPN1* | -3.03303 | 4.17E-07 | *HSD11B2* | -1.4346 | 3.93E-05 | *PPFIA3* | -1.02554 | 0.000432782 | *ZDHHC23* | -1.08139 | 0.000458252 |
| *CR2* | -2.61705 | 0.000596629 | *HTR7* | -1.93014 | 3.46E-10 | *PPFIBP2* | -1.41845 | 1.57E-09 | *ZFPM2* | -1.3769 | 2.96E-05 |
| *CRABP1* | -1.0435 | 5.88E-06 | *HTR8* | -1.67234426 | 1.84E-03 | *PPM1N* | -1.1798 | 0.00332366 | *ZIC3* | -1.45865 | 2.63E-09 |
| *CRLF1* | -1.34857 | 2.28E-05 | *HTR9* | -1.425961403 | 2.82E-03 | *PPP2R2C* | -1.95674 | 2.91E-09 | *ZNF204P* | -1.16354 | 1.83E-05 |
| *CRYM* | -1.3051 | 0.00262012 | *ID1* | -1.0495 | 9.97E-06 | *PPP2R2C* | -1.721620831 | 1.65E-03 | *ZNF578* | -1.12151 | 7.68E-05 |
| *CSRP3* | -2.53397 | 0.00108805 | *IFLTD1* | -3.42259 | 1.09E-09 | *PPP2R2C* | -1.475237974 | 2.63E-03 | *ZNF804A* | -1.12067 | 0.00254669 |
| *CST1* | -4.35994 | 0 | *IGFBP6* | -2.48307 | 0 | *PRAP1* | -2.9329 | 3.67E-07 | *ZYG11A* | -1.46454 | 0.000184669 |
| *CST2* | -3.80015 | 0 | *IGFL3* | -3.65771 | 3.11E-05 | *PRKCG* | -1.1282 | 0.00192101 |  |  |  |
| *CST4* | -3.45072 | 0 | *IGSF1* | -1.02528 | 0.00588229 | *PRKCQ* | -1.18546 | 4.80E-05 |  |  |  |
| *CTCFL* | -2.32833 | 0.00247863 | *IGSF5* | -1.52084 | 0.00356986 | *PRKCQ-AS1* | -1.00704 | 0.00695302 |  |  |  |
| *CTH* | -1.06805 | 7.83E-05 | *IGSF9B* | -1.08941 | 0.000676404 | *PROKR1* | -1.41741 | 0.00301116 |  |  |  |
| *CUBN* | -1.09872 | 0.000508169 | *IHH* | -3.17892 | 0 | *PROM1* | -1.11145 | 7.63E-07 |  |  |  |
| *CXCL5* | -1.13989 | 0.00108308 | *IL6R* | -1.14792 | 0.00266328 | *PRSS1* | -3.95317 | 5.15E-05 |  |  |  |
