# Supplementary material for: Transcriptional Profiling of Ectoderm Specification to Keratinocyte Fate in Human Embryonic Stem Cells
Source: PLoS One. 2015 Apr 7;10(4):e0122493. doi: 10.1371/journal.pone.0122493 (PMC4388500; doi:10.1371/journal.pone.0122493)
Supplement: S3 Table — (DOCX) [file pone.0122493.s003.docx]

**S3 Table. List of *Homo sapiens* primers used for qRT-PCR analysis.**

| *Homo sapiens* | | |
| --- | --- | --- |
| Gene | Fw primer | Rev primer |
| *KERATIN 18* | TGAGACGTACAGTCCAGTCCTT | GCTCCATCTGTAGGGCGTAG |
| *KERATIN 14* | TCAGCATGAAAGCATCCCTGGAGAA | ATTTGGCGGCTGGAGGAGGTCA |
| *TRP63* | AGCCAGAAGAAAGGACAGCAGCATT | CTGTGCGGGCCTGGGTAGTC |
| *OCT4* | CCCCTGGTGCCGTGAAGCTG | CCCCAGGGTGAGCCCCACAT |
| *AP2α* | GCATATCCGTTCACGCCGAT | GGGAGATTGACCTACAGTGCC |
| *DLX3* | CTTACTCGCCCAAGTCGGAAT | AGTAGATCGTACGCGGCTTTC |
| *IRF6* | AGCGTGGTAGCGACGGGTGA | CGGGGGTGGAGGGCCATGAT |
| *PAX6* | GCCCTCCGCTCCCAGGAATCT | CACATCTGCGCGCCCCTAGT |
| *FGF19* | AGATCAAGGCAGTCGCTCTG | CGGATCTCCTCCTCGAAAGC |
| *BMP10* | ATGCCCTCTGCCAACATCAT | GGAGGGGGTATTTTCGGAGC |
| *EGF* | TGTCCCTTTTTGGTGACCGTAT | CAAAGTTTCTGCTCAGGCTCC |
| *TGM1* | CTCTGGCACTCGAAGACCTG | TACTAGCATGCCCTCTCGGA |
| *BNC1* | CCGAGGCTATCAGCTGTACT | GGTCCCATAGAGCATGAGGC |
| *BARX2* | CCAGACAGGTTGGACTTGGCTCAG | GGGCTCCAGCTGCTCCTGAC |
| *LHX1* | CTCATCAGACCAAACCAAAGACCAT | CCAGGTCGCTAGGGGAGATG |
| *NFATC1* | GCATCACAGGGAAGACCGTGTC | GAAGTTCAATGTCGGAGTTTCTGAG |
